# Interplay of the ENS and Microbiota With Murine Gut Epithelium‐Derived Organoids in Aging

**DOI:** 10.1111/acel.70608

**Published:** 2026-07-03

**Authors:** Tinh Thi Nguyen, Dennis Schapelhouman, Katharina Fischer, Jia‐Xuan Chen, Mario Dejung, Fridolin Kielisch, Julia Varga, Johannes Piepgras, Matthew Ahn, Oliver Tüscher, Peter Baumann, Sandra Schick, Kristina Endres

**Affiliations:** ^1^ Department of Psychiatry and Psychotherapy University Medical Center Mainz Mainz Germany; ^2^ Chromatin Regulation Group Institute of Molecular Biology Mainz Germany; ^3^ Faculty of Computer Sciences and Microsystems Technology Kaiserslautern University of Applied Sciences Kaiserslautern Germany; ^4^ Institute of Molecular Biology Mainz Germany; ^5^ Leibniz Institute for Resilience Research Mainz Germany; ^6^ The University Clinic and Polyclinic for Psychiatry, Psychotherapy and Psychosomatics University Medical Center Halle Halle Germany; ^7^ Department of Biology Johannes Gutenberg University Mainz Germany

**Keywords:** aging, enteric nervous system, epithelium, gut, microbiota, organoid

## Abstract

The intestine is one of the first organs to show signs of aging, including cellular changes, microbiota shifts, and reduced regenerative capacity. The different components of the gut—such as the epithelium (which is directly exposed to a diverse array of host–microbe interactions), the microbiota itself, and the underlying enteric nervous system—likely contribute to aging in distinct ways. Understanding their individual and interactive roles is key to elucidating the mechanisms of intestinal aging. To better understand the contribution of individual components to intestinal aging, we analyzed gut tissue characteristics and compared these parameters with the composition and gene expression levels of colonic organoids by using two mouse strains: the aging‐resistant SAMR1 line and the SAMP8 line, which exhibit an accelerated aging phenotype. Here, we demonstrate that colonic organoids derived from these mice retain the age‐related characteristics of the colonic tissue, including changes in morphology and cellular composition. Furthermore, introducing the enteric nervous system into organoid culture revealed that the age of the epithelium exerts a more pronounced influence on the aging phenotype than the age of the innervating tissue. Interestingly, successfully delivering fecal extracts to organoids revealed that gut microbiota metabolites from aged animals resulted in an aging phenotype of the gut epithelium in vitro. In summary, our findings indicate the impact of aging on the gut epithelium and its interplay with the nervous system and microbiota. This may in future provide new strategies for slowing the aging process in the gut by manipulating the gut commensals.

## Graphical abstract

A gut organoid model was developed to study the impact of age‐related changes of the enteric nervous system (ENS) and microbiome on the integrity and trophic capacity of the intestinal epithelial barrier. Organoids reflected in vivo aging features, including altered epithelial integrity and changes in acetylcholine‐related enzyme activity. Fecal water from aged mice induced aging traits in organoids derived from young animals, validating the model for aging studies.

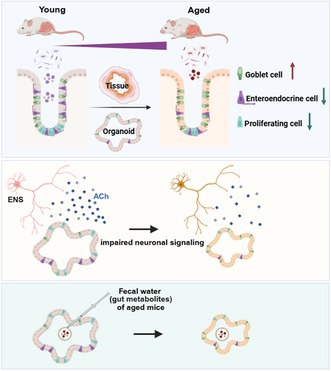

## 1 Introduction

Aging is characterized by the progressive dysfunction of most tissues and organs, which has been linked to a gradual decline in regenerative capacity, cell proliferation, telomere maintenance, and genome stability. This deterioration is the main risk factor for significant human pathologies, such as cancer, diabetes, cardiovascular disorders, and neurodegenerative diseases (Tartiere et al. 2024).

The gastrointestinal (GI) tract plays a key role in digestion, nutrient and drug absorption, and protection against ingested pathogens (Ogobuiro et al. 2024). In order to perform its physiological functions, the gut requires the coordination of its various components, including the intestinal epithelial barrier, which is connected by connective tissue, as well as the immune system, the enteric nervous system (ENS), and the vascular system. All of these components are distributed across the different layers of the intestinal wall. Furthermore, the GI tract harbors a complex and diverse microbiota that plays an important role in metabolism, immune functions, and the regulation of host health.

As we age, the components of the gut undergo significant changes. Aging is associated with defects in mucosal defense function and structure, as well as changes in the differentiation rate of intestinal epithelial cells, which alter gut structure and physiology (Suzuki et al. 2022). Aging also leads to structural and functional alterations in the ENS, including a decline in neuron density, neurotransmitter dysregulation, and impaired gut motility, which contribute to GI dysfunction (Nguyen et al. 2023). Dysbiosis, decreased microbiota diversity, and a higher proportion of proteolytic bacteria, which influence microbiota‐derived metabolites, may all contribute to the increasing prevalence of many age‐related diseases in the elderly (Ragonnaud and Biragyn 2021). Investigating these factors individually in a living organism is challenging due to their complex interconnections. It remains unclear which gut components primarily drive aging‐related changes, highlighting the need for models that can isolate specific factors to identify the key drivers of aging.

Methods for culturing three‐dimensional gut tissue were described, including the use of colonic organoids (Sato et al. 2009), which provide an organotypic structure in vitro and offer a valuable alternative to animal research. However, a study of aging phenotypes in the gut using organoids is insufficient due to the limitation that organoids exclusively represent the gut epithelium (Jo et al. 2023). Only a few studies have examined the co‐culture of intestinal organoids and the ENS using models of induced human pluripotent stem cells and mice (summarized in Llorente 2024), but these models have not considered aging. While Mishra et al. (2024)'s research examined the impact of microbiota metabolites on gut permeability and inflammation, this approach does not replicate direct interactions with the gut lumen. To our knowledge, no comprehensive study has yet assessed the impact of aging on both the gut epithelium and the ENS in a culture model. Nor has there been an examination of the direct impact of aging on microbiota interactions with the gut epithelium.

The present study aims to investigate how the gut tissue changes during aging and to define the unique contributions of the ENS and microbiota to gut aging. To this end, we used two models of aging: senescence‐accelerated aging mice and normal aging mice. We compared the intestinal cellular heterogeneity and intestinal functionality of young (3 months old) and aged (10 months old) animals. We found that age‐related phenotypes are preserved in colonic organoids in terms of cellular composition. Next, we evaluated the effect of aging on the interaction between organoids and the ENS in a co‐culture system. Subsequently, we introduced microbiota‐derived metabolites (fecal water, FW) to assess their impact on organoid culture using microinjection. Our study revealed that it is organoid age rather than ENS age that drives age‐related changes in organoid growth and ENS activities. It also highlights that microbiota‐derived metabolites from an aged organism impact gut epithelial organoids and can introduce an aged phenotype.

## 2 Materials and Methods

### 2.1 Mice

Both male and female SAMP8 (senescence‐accelerated mouse prone 8) and SAMR1 (senescence‐accelerated mouse resistant 1) control mice aged 3 and 10 months, originating from Envigo (Indianapolis, IN, USA), were bred under a license in the Translational Animal Research Center of Johannes Gutenberg University. Mice aged 3 months were designated as “young”, whereas mice aged 10 months were designated as “aged”. The mice were maintained on a 12 h light/dark cycle and provided with food (ssniff Spezialdiäten, Soest, Germany) and water ad libitum. Animals were kept in groups of no more than five per cage. All procedures were performed in accordance with the European Communities Council Directive on the care and use of animals for experimental procedures and were approved by local authorities (Landesuntersuchungsamt Rhineland‐Palatinate).

### 2.2 Preparation of Gut Tissue for Staining

Mice were anesthetized with isoflurane and then sacrificed. Colon samples (1 cm from the proximal end) were collected immediately after dissection. The samples were flushed with phosphate buffered saline (PBS) and fixed in ROTIHistofix (Carl Roth) for 24 h at 4°C. The samples were then dehydrated and embedded in paraffin wax. A 5 μm thick section of the formalin‐fixed paraffin‐embedded (FFPE) block of gut tissue was cut into sections using the Leica Rotatory Microtome RM2255.

### 2.3 LMMP Preparation and Culture

The colon was collected and rinsed with gassed Krebs solution (118 mM NaCl, 4.6 mM KCl, 1.3 mM NaH_2_PO_4_, 1.2 mM MgSO_4_, 25 mM NaHCO_3_, 2.5 mM CaCl_2_, and 10 mM glucose). The tissue was then mounted over a rod, and the entire mesenteric attachment line was gently rubbed with the edge of the forceps and a cotton swab to separate the longitudinal muscle with the attached myenteric plexus (abbreviated as LMMP) from the underlying circular muscle. The LMMP was then teased away from the circular muscle using a cotton swab wetted with Krebs solution. The collected LMMP tissue was cut into small pieces and kept in gassed ice‐cold Krebs solution until the next step. The LMMP tissue pieces were transferred on droplets of basement membrane extract (10 μL BME; R&D Systems, 3533‐010‐02) in the wells of a 24‐well suspension plate (Greiner Bio‐One). The BME was polymerized in an incubator at 37°C with 95% humidity, and 5% CO_2_ for 10 min. Then, cENR medium consisting of advanced DMEM/F12 (Gibco, 12634‐010) supplemented with 1 × GlutaMAX (Thermo Fisher Scientific, 35050‐038), 100 U/mL Penicillin–Streptomycin (Thermo Fisher Scientific, 15140122), 10 mM HEPES (Thermo Fisher Scientific, 15630‐056), 1 × B‐27 (Thermo Fisher Scientific, 17504044), 1 × *N*‐2 (Thermo Fisher Scientific, 17502‐048), 10% (*v*/*v*) Noggin conditioned medium (see Supporting Information), 20% (*v*/*v*) R‐spondin conditioned medium (see Supporting Information), 50 ng/mL mouse EGF (Peprotech, 315‐09‐100), 1.25 mM N‐acetyl‐L‐cysteine (Sigma‐Aldrich, A9165), 0.5 μM A83‐01 (Sigma‐Aldrich, SML0788), and 3 μM CHIR99021 (MedChemExpress, CAY13122‐5) was added to the wells. The plates were then incubated at 37°C with 95% humidity and 5% CO_2_ for 7 days. The medium was changed every 3 days. Afterwards, after cells migrated from the thin tissue layer onto the culture vessel surface, the LMMP strip was removed. Thus, the method is referred to as a stamp‐like approach.

### 2.4 Establishment of Colonic Organoid Culture

The colon was opened longitudinally, cut into pieces measuring 2–4 mm, and transferred to a tube containing ice‐cold PBS. The pieces were then washed eight times with cold PBS before being incubated in 5 mM EDTA in DPBS at 4°C for 45 min. Afterwards, the supernatant was removed and replaced with ice‐cold AD‐DF+++ (Advanced DMEM/F12 supplemented with 1 × GlutaMAX, 100 U/mL Penicillin–Streptomycin, 10 mM HEPES). The intestinal crypts were subsequently isolated by vortexing for 3–5 s at level 7 (Vortex REAX 2000) and by repeated pipetting. They were then collected through a 100 μm cell strainer (Greiner Bio‐One) and spun down at 800 × *g* for 5 min at room temperature (RT). The crypt pellets were resuspended in 10 mL of ice‐cold AD‐DF+++. The number of crypts was calculated by counting the number of crypts in 10 μL of the crypt suspension, using an inverted microscope, and then multiplying this number by 1000 to give the total number of crypts per mL. The isolated crypts were seeded in BME (500 crypts in a 50 μL BME dome per well) and cultured in cENR medium. For the first three days following seeding, the medium was supplemented with 10 μM Y‐27632 (Biomol, CAY10005583‐10). The medium was completely changed every 3 days. The resulting organoids were passaged every 7 days.

### 2.5 ENS and Organoid Co‐Culture

Female young and aged SAMR1 mice were used for the co‐culture of ENS and organoids. To obtain ENS cells, LMMPs were first cultured as described above. On day 7, the LMMPs were removed with forceps and the remaining cells migrating from LMMP explants were grown within the BME dome until day 14. The ENS cells were then used for immunofluorescent staining and co‐cultured with organoids on day 14. After LMMP removal, migrated ENS cells were co‐cultured with isolated crypts or passaged organoids in cENR medium in a 37°C incubator with 95% humidity and 5% CO_2_ for 7 days. Half of the medium was changed every 3 days.

### 2.6 Preparation of FW

Feces were collected from young and aged female SAMR1 mice (*n* = 5 each). An equal amount of feces from each mouse of the same age was pooled together. The protocol for FW preparation was performed as previously described (Klinder et al. 2007). Briefly, fecal slurry was prepared by mixing feces with ice‐cold PBS and homogenizing it for 3 min (1 g feces per mL PBS). The homogenates were then centrifuged at 35,000 × *g* for 2 h at 4°C, and the resulting supernatant was collected. These were then filtered through a 0.22 μm filter (Cytiva Whatman) to obtain the FW, which was then aliquoted and stored at −80°C.

### 2.7 Injection of Organoids With FW

The organoid injection protocol was adapted from a previously published protocol (Engevik et al. 2018). Organoids derived from five independent young female SAMR1 mice (*n* = 5) on day 4 after passage were used for the injection of FW. The organoids were seeded at a density of 20–30 organoids per BME dome, with two BME domes per well and two wells per condition to obtain sufficient material for downstream analysis. Each organoid received a single injection. FW was injected into the organoids at a volume of 50 nL and a speed of 25 nL/s using a Nanoject III microinjector (Drummond Scientific), while PBS served as a solvent control for FW. To confirm the successful injection, 5 mg/mL of propidium iodide (Sigma) was used (see Figure S1). Following injection, the organoids were monitored in culture until day 7, after which they were collected for RNA isolation.

### 2.8 Organoid Sample Collection

To obtain the organoid sample, the organoids were collected in Cell Recovery Solution (Corning) and incubated on ice for 30 min. The organoid suspension was then centrifuged at 400 × *g* for 5 min. The organoids were washed in cold PBS and centrifuged again at 400 × *g* for 5 min.

For biochemical analysis, the organoid pellets were washed again in cold PBS and centrifuged at 400 × *g* for 5 min. The organoid pellets were then stored at −80°C for RNA and protein isolation.

For the histological analysis, the organoid pellets were resuspended in 4% paraformaldehyde in PBS, fixed for 16 h at 4°C, and then washed twice in cold PBS. The samples were subsequently dehydrated in 70% ethanol (Carl Roth). After removing the ethanol, liquefied Epredia Richard‐Allan Scientific HistoGel (Thermo Fisher Scientific, HG‐4000‐012) was added to the organoid samples, which were then transferred to a 10 × 10 × 5 mm tissue‐tek cryomold (Sakura) and allowed to solidify at RT. The solid HistoGel plug was then removed from the cryomold and placed in a biopsy cassette (Simport). The samples were stored in 70% ethanol in a refrigerator until processing. The organoids were then dehydrated using ascending grades of alcohol (70%, 80%, 96%, and 100% ethanol, and then 100% ethanol again) and twice in Roticlear (Carl Roth), using a Leica TP1020 tissue processor. Finally, the organoids were embedded in paraffin (Leica, 39602004). The FFPE block of colon organoids was cut into 5 μm sections using the Leica RM2255 Rotatory Microtome.

### 2.9 H&E Staining of Gut Tissue and Crypt Depth Analysis

The tissue sections were incubated at 60°C for 30 min, then dewaxed in xylene (Carl Roth), and subsequently rehydrated using a series of graded alcohol solutions (ethanol at 100%, 96%, and 70%). The sections were stained with hematoxylin and eosin (H&E) according to the standard procedure. They were then dehydrated using ascending concentrations of ethanol (70%, 96%, and 100%) and cleared using xylene. Three images were captured per mouse using the EVOS XL Core microscope (Thermo Fisher Scientific).

The crypt depth was analyzed using ImageJ software (https://imagej.net/ij/). Images captured using a 4× objective lens were divided into four quarters by one vertical and one horizontal line. The first intact crypt close to such a line in each quarter was measured in a clockwise direction to avoid bias when selecting structures for analysis.

### 2.10 Alcian Blue Staining

Mucin is one of the main proteins produced by the gut epithelium and forms the mucus barrier that faces the gut lumen. To quantify mucin‐producing goblet cells and mucin abundance in fixed gut tissue and colonic organoids, samples were stained with alcian blue which stains structures containing acidic mucins. The FFPE slides were first deparaffinized and rehydrated by immersing them in xylene and then in a series of descending concentrations of ethanol (100%, 95%, 70%, and 50%), followed by deionized water. The slides were then stained in an alcian blue solution (Morphisto) for 30 min at RT. They were then washed in running tap water for 2 min and rinsed in distilled water. Next, the slides were counterstained in nuclear fast red solution (Sigma‐Aldrich) for 10 min, followed by a final wash in running tap water for 1 min. The slides were dehydrated by immersing them in a series of alcohol solutions (95% and twice 100%). Finally, the slides were then cleared in xylene and the tissue was mounted with a resinous mounting medium (Sigma‐Aldrich). Images were taken using the EVOS XL Core microscope and the intensity was measured using Fiji software (http://fiji.sc).

### 2.11 Immunofluorescent Staining

The FFPE slides containing gut tissue and colon organoids were deparaffinized and dehydrated as described above. For antigen retrieval, the slides were boiled in 10 mM sodium citrate buffer pH 6.0 for 20 min in a microwave. The slides were then left to cool in the buffer for 20 min at RT, after which they were washed again by immersing them in PBS for 5 min. The sections were permeabilized for 10 min with 0.2% Triton X‐100 (Carl Roth) diluted in PBS and then washed twice with PBS for 5 min. After that, the slides were incubated in blocking solution (Cell Signaling, 12411S) for 1 h at RT. The slides were washed again in PBS for 5 min. The sections were then incubated for overnight at 4°C with primary antibodies anti‐Chga (chromogranin A; Santa Cruz, sc‐393941, dilution 1:100) and anti‐Ki67 (Antigen Kiel 67; Cell Signaling, 9129T, dilution 1:400) in antibody dilution buffer containing 1% BSA and 0.3% Triton X‐100 in PBS. After four further washes with PBS for 5 min each, the samples were incubated with the secondary antibody conjugated with either AF594 (anti‐rabbit IgG Fab2, Alexa Fluor 594, Cell Signaling, 8889S, dilution 1:500) or AF488 (anti‐mouse IgG Fab2, Alexa Fluor 488, Cell Signaling, 4408S, dilution 1:500) in antibody dilution buffer for 1 h at RT, followed by four more washes with PBS for 5 min each. The slides were then incubated with 0.5 μg/mL DAPI (Sigma‐Aldrich) in PBS. After a final four washes with PBS, the slides were dried and the tissues were covered with mounting medium (Vectashield, H1000). Images were taken from each slide by using the microscope unit of the Single Cellome System 2000 (Yokogawa) for analysis, and the fluorescent intensity was quantified using Fiji software.

### 2.12 RNA Isolation and RT‐qPCR

RNA was isolated from the harvested organoids (as described above) using a TRIzol (Qiagen) extraction followed by a cleanup using the RNeasy Mini kit (Qiagen) and treatment with RNase‐free DNase (Qiagen), according to the manufacturer's instructions. RT‐qPCR was conducted using the QuantiNova SYBR Green RT‐PCR Kit (Qiagen) according to the manufacturer's instructions on a StepOnePlusReal‐Time PCR System (Applied Biosystems). Relative mRNA expression, normalized to *Gapdh* expression, was calculated using the ΔΔCt method (see the primer list in Table S1).

### 2.13 Protein Extraction and Proteomics Analysis

The harvested organoids were homogenized in RIPA buffer (25 mM Tris–HCl, pH 8, 1 mM EDTA, 150 mM NaCl, 1% Nonidet P‐40, 0.5% sodium deoxycholate, 0.1% SDS, and 1× proteinase inhibitor cocktail) using a Bioruptor Diagenode Plus (B01020001) at 30 s ON and 30 s OFF for 10 cycles. The homogenized samples were spun down at 14,000 × *g* for 15 min at 4°C (HERMLE Z 216 MK, angle rotor 220.87), and the supernatant was collected. Protein concentration was measured using the Bradford assay. 50 μg of protein were used for proteomics processing (see Supporting Information).

Scatterplots indicated high reproducibility within each batch but also a batch effect (see Figures S2 and S3). Additionally, the batch effect was observed in a principal component analysis (PCA) of the 500 most variable proteins (see Figure S4). Although median normalization reduced this effect to some extent, it did not fully remove it. To account for residual batch‐related variation, batch was included as a covariate in the linear model used for downstream statistical analyses. The full model included ‘genotype’, ‘age’, ‘sex’, and ‘batch’ as explanatory variables. Model selection was guided by the second‐order Akaike information criterion (AICc). An advantage of this approach is that it identifies not only the best‐supported model for each protein but also alternative models with substantial support. This enabled selection of a common model that was supported by the majority of proteins and was biologically plausible. In the final model, genotype and age were specified as interacting effects, whereas sex and batch were included as additive effects without interaction terms (R formula: log2_int_norm~genotype × age + sex + batch). After filtering for proteins with a complete, balanced design (quantified in two biological replicates per combination of ‘genotype’, ‘age’, and ‘sex’), 7066 proteins out of 7545 proteins were used for differential expression analysis (filter for at most one missing value out of the 32 samples). Statistical analysis was carried out with the R‐package ‘limma’ version 1.47.5 (Ritchie et al. 2015) and ‘MuMIn’ version 1.47.5 (DOI: 10.32614/CRAN.package.MuMIn), fitting empirical Bayes moderated linear models.

### 2.14 Western Blotting

Western blotting was performed according to a previous publication (Rai et al. 2026). For the pre‐test to validate the antibodies against Fut2 and Fut4, murine tissues with known expression status, as well as colonic organoids treated with fucose (substrate of either enzyme), were analyzed (see Supporting Information). For the main experiments, briefly, 25 μg of protein extracted from organoids as described above was loaded onto a 10% SDS‐PAA gel and transferred to a nitrocellulose membrane by tank blot method. The primary antibodies were as follows: anti‐GAPDH (Cell Signaling, 2118, dilution 1:1000 in 0.2% I‐Block blocking solution (Thermo Fisher Scientific)), anti‐Fut2 (Biorbyt, orb 156968, dilution 1:1000 in 5% nonfat dried milk blocking solution (ITW Reagents)), and anti‐Fut4 (Biorbyt, orb627154, dilution 1:1000 in 5% nonfat dried milk blocking solution (ITW Reagents)). Quantification was performed by using appropriate HRP‐conjugated secondary antibodies and SuperSignal West Femto Maximum Sensitivity Substrate (Thermo Fisher Scientific), with signal acquisition carried out using a Lumi Imager F1 (Boehringer Mannheim).

### 2.15 Single‐Clonal Organoid Culture With Incucyte

The organoids were collected by dissociating BME in AD‐DF+++ medium using a pipette for 2 min. The cell suspension was spun down at 500 × *g* for 5 min in an Eppendorf 5804 centrifuge, after which the supernatant was discarded. The cell pellets were resuspended in Accutase solution (Sigma‐Aldrich, A6964) for 5 min at a 37°C water bath. Then, AD‐DF+++ medium was added, after which the cells were spun down again at 800 × *g* for 5 min. The cells were resuspended in AD‐DF+++ medium and filtered through a 40 μm cell strainer (Greiner Bio‐One). We counted viable cells (Trypan Blue (Gibco) negative), using a DeNovix CellDrop FL cell counter. The cells were seeded at a density of 35,000 cells/well in a 96‐well plate (Corning), embedded in BME and cultured with cENR medium containing 10 μM Y27632. The plates were placed in a CO_2_ incubator at 37°C with 95% humidity for one day before being transferred to an Incucyte SX5 (Sartorius). The medium was changed every 3 days. The Incucyte microscope took images of each well every 6 h for 6 days, after which the total organoid area was analyzed using the Incucyte software.

### 2.16 Transepithelial Electrical Resistance (TEER) Measurement

Organoids derived from female SAMR1 and SAMP8 mice aged 10 months (*n* = 5) were used. Before seeding, Millicell 24 well hanging cell culture inserts (Merck) were coated with 5% BME diluted with ice‐cold AF‐DF+++ medium for 2 h in a CO_2_ incubator at 37°C with 95% humidity. Once coating was complete, excess BME solution was aspirated before seeding the cells. 2D organoid‐derived monolayers were generated from the 3D organoid cultures. The organoids were collected by dissociating BME in AD‐DF+++ medium using a pipette for 2 min. The resulting cell suspension was centrifuged at 500 × *g* for 5 min in an Eppendorf 5804 centrifuge, after which the supernatant was removed. The cell pellets were resuspended in Accutase solution supplemented with 10 μM Y27632 and incubated for 5 min in a 37°C water bath. Subsequently, pre‐warmed AD‐DF+++ medium containing 10 μM Y‐27632 was added, and the cells were passed through a 40 μm cell strainer, followed by centrifugation at 800 × *g* for 5 min. Cells were counted using a Scepter 3.0 Cell Counter (Merck) and seeded into BME‐coated transwell inserts at a density of 80,000 cells/well. Cells were added to the apical chamber of BME‐coated transwells in 300 μL of cENR medium containing 10 μM Y27632 and 700 μL of cENR medium containing 10 μM Y27632 was added to the basolateral chamber. Medium was changed every 3 days. After seeding, organoid‐derived monolayers were grown for 10 days, after which they were switched to differentiation medium (AF‐DF+++ medium supplemented with 1 × B‐27, 1 × *N*‐2, 10% (*v*/*v*) Noggin conditioned medium, 50 ng/mL mouse EGF, and 1.25 mM N‐acetyl‐L‐cysteine) for 3 days prior to TEER measurement. Barrier integrity of cell monolayers was evaluated by measuring TEER using a Millicell ERS 3.0 Digital Voltohmmeter (Merck) according to the manufacturer's instructions. The resistance values obtained (Ω) were converted to TEER by multiplying by the surface area of the transwell inserts (0.3 cm^2^). For monolayers derived from SAMP8 mice, TEER measurements were additionally normalized to those from SAMR1‐derived samples and expressed as percentage relative to control (CTR).

### 2.17 Enzymatic Activity Assays

The LMMP samples were collected from cultures in the absence and presence of organoids and homogenized as previously described (Stoye et al. 2020). Briefly, the activity of acetylcholine esterase (AChE) was measured using 1 μL of the lysate supernatant (diluted to 10 μL/mg tissue in potassium phosphate buffer). Choline acetyltransferase (ChAT) activity was quantified according to the manufacturer's instructions (Elabscience, TX, USA) using 10 μL of tissue homogenate.

### 2.18 Statistical Analysis

The data were analyzed using Graphpad Prism version 8 software (https://www.graphpad.com/). All graphical data are presented as mean ± SEM. Statistical significance for the comparison of TEER measurement and organoid forming efficiency was determined using an unpaired two‐tailed Welch's *t*‐test and two‐way ANOVA followed by Tukey's post hoc test, respectively. Other comparisons were analyzed using one‐way ANOVA followed by Sidak's multiple comparisons test or Fisher's LSD post hoc test. The statistical significance thresholds for comparisons between genotypes and ages were set as follows: *p < 0.05, **p < 0.01, and ***p < 0.001. Detailed definitions of replicates and statistical information for each panel are provided in Table S2.

## 3 Results

### 3.1 Age‐Related Changes of Colonic Tissue Are Preserved in Colonic Organoids

Our aim was to establish a toolbox for investigating the interplay of gut composites along the aging process, specifically the epithelium that is in contact with the ENS and the microbiota. Firstly, we had to prove that colon organoids, as ex vivo representatives of the gut epithelium, reflect an aging phenotype resembling that of the corresponding donor animal. To achieve this, we compared the cellular characteristics of colonic tissue and colonic organoids from two mouse strains at young and old age: SAMR1 mice were used as a strain exhibiting healthy aging, while SAMP8 mice were used as a model of accelerated aging. We defined 3 and 10 months of age as ‘young’ and ‘aged’, respectively, based on behavioral testing of the mice (data not shown), which revealed that the two mouse strains were relatively comparable at 3 months. Behavioral and phenotypic differences such as fur condition evolved at 5 and 7 months of age and were manifested at 10 months of age. Moreover, SAMP8 mouse mortality rates increased from 10 months of age onward. For the sake of 3R, therefore, this age was defined as the endpoint of our study.

**FIGURE 1:**
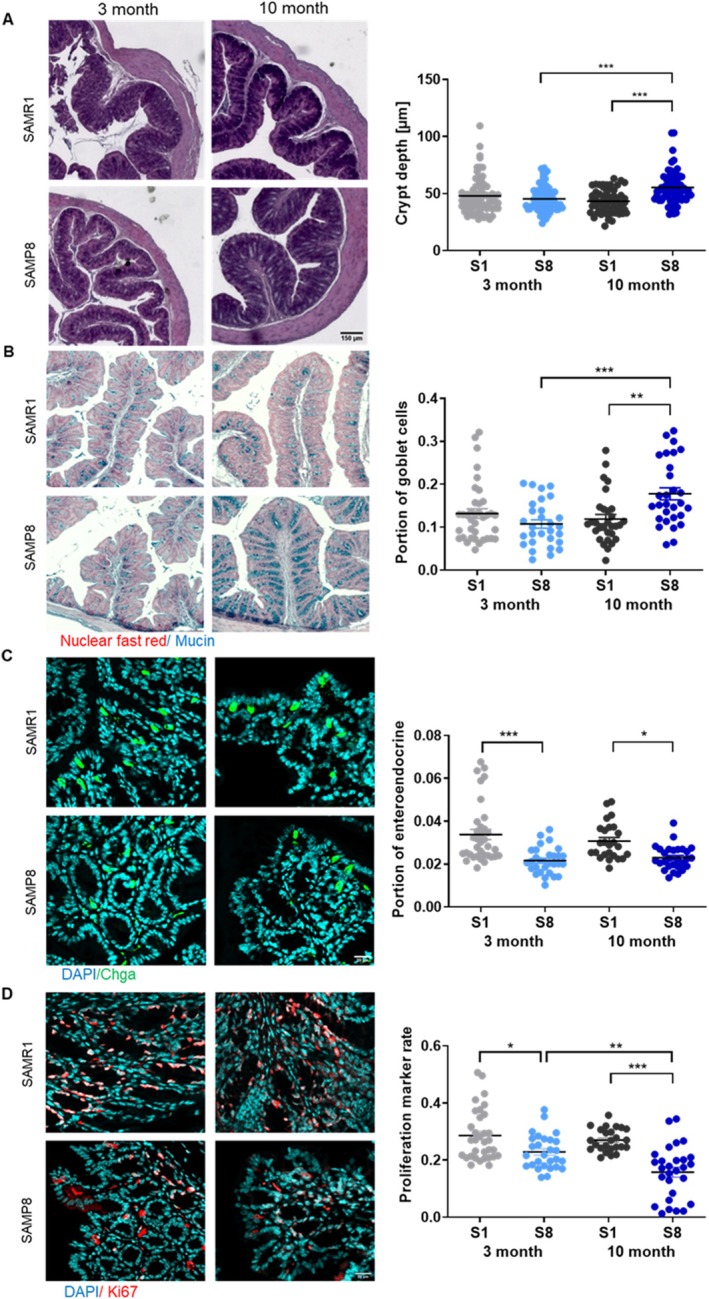
Age‐related changes in gut morphology and cellular compositions in the colon of SAMR1 and SAMP8 mice. Gut tissue samples were isolated from SAMP8 and SAMR1 mice at 3 and 10 months of age (*n* = 3 animals for each combination of genotype, sex, and age; five image fields per animal were analyzed; individual data points represent single measurements). (A) Hematoxylin and eosin (H&E) staining of gut tissue to measure crypt depth. Scale bar: 150 μm. (B) Mucin staining using alcian blue as an indicator of goblet cells (blue) and nucleus staining using nuclear fast red (pink). (C) Immunofluorescence co‐staining of chromogranin A (Chga, green) as an enteroendocrine cell marker and nuclei with DAPI (cyan). (D) Immunofluorescence co‐staining of Ki67 (red) as a proliferation marker and nuclei with DAPI (cyan) and quantification of the proportion of Ki67‐positive cells relative to total cell number (DAPI‐positive nuclei). Scale bar: 20 μm. Values are presented as mean ± SEM. Statistical analysis was performed by one‐way ANOVA with Sidak's post‐test; *p < 0.05, **p < 0.01, ***p < 0.001 (Table S2). Abbreviations: S1, SAMR1; S8, SAMP8.

Histological analysis of H&E‐stained colon sections revealed crypt elongation in aged SAMP8 mice compared to young SAMP8 and aged SAMR1 mice (Figure 1A, Table S2). This seems consistent with chronic inflammation as crypt hyperplasia has been described to occur at least in small intestinal inflammatory conditions (Arato et al. 1994; Stamnaes et al. 2025; Toritani et al. 2022). Next, the colon sections were stained for different epithelial cell markers such as mucin as a marker for goblet cells, Chga as a marker for enteroendocrine cells (EECs), and Ki67 as a marker for proliferation (Figure 1B–D). The number of goblet cells increased with age in SAMP8 mice, particularly in the aged group, compared to SAMR1 (Figure 1B). Expression of the EEC marker was found decreased in colon tissue of young SAMP8 mice compared to SAMR1 mice as well as in colon tissue of aged SAMP8 mice compared to aged SAMR1 mice (Figure 1C). Furthermore, expression of the proliferation marker was reduced with age in SAMP8 mice, as well as in both age groups of SAMP8 mice, compared to SAMR1 mice (Figure 1D, Table S2). Together, these results demonstrate that age‐related alterations occur not only in the morphology but also in the cellular composition of the gut.

**FIGURE 2:**
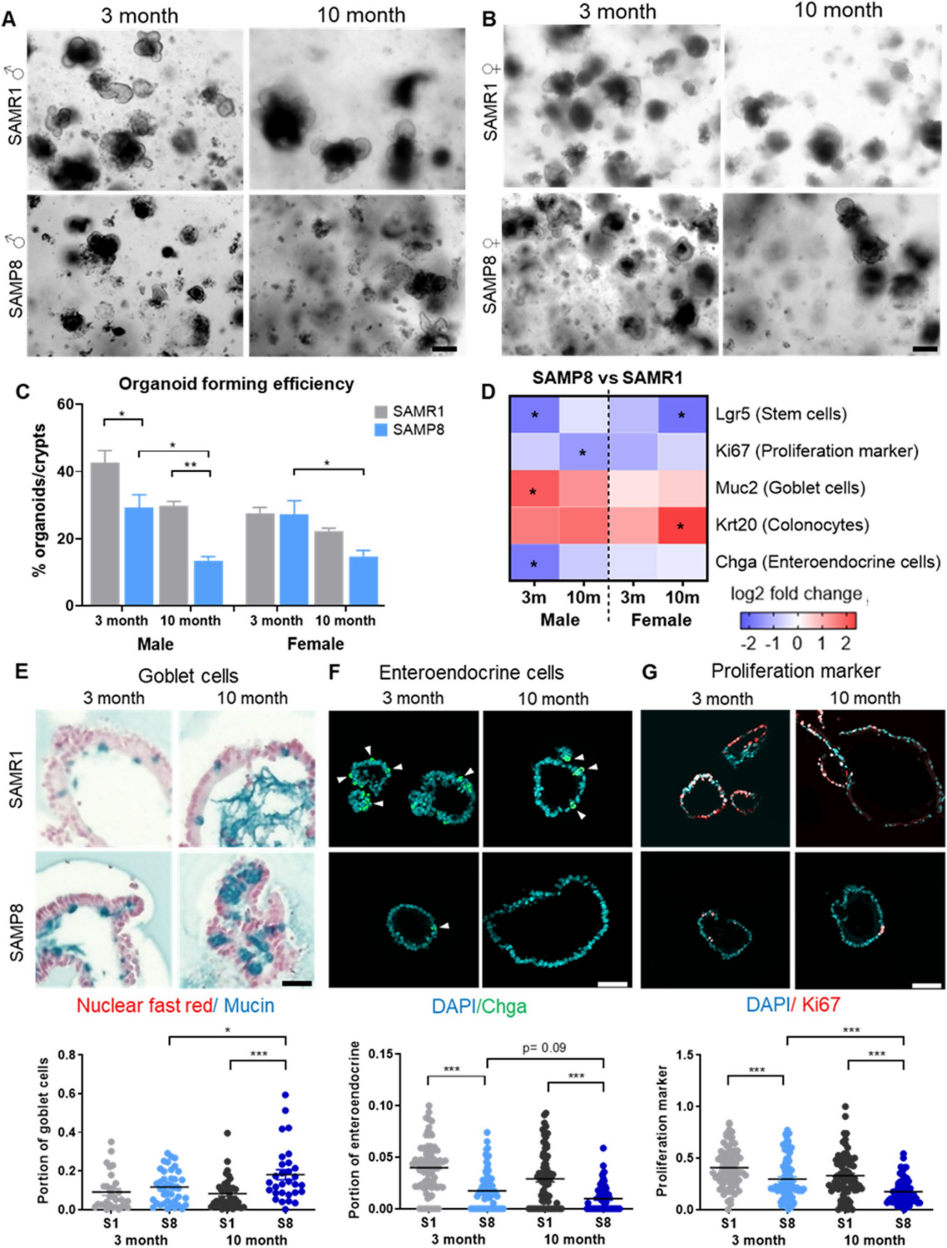
Characterization of colonic organoids derived from SAMP8 mice compared to SAMR1 mice. Organoids were assessed in terms of growth capacity and cell compositions. Representative images of organoids derived from male (A) and female (B) SAMR1 and SAMP8 mice at day 7 in vitro. Scale bar: 200 μm. (C) The organoid forming efficiency between groups regarding age and strain difference, which is calculated as the number of formed organoids at Day 7 divided by the total number of initially isolated crypts at Day 0, multiplied by 100 (*n* = 5 animals per genotype, per sex, per time point). Statistical analysis was performed by two‐way ANOVA followed by Tukey's post hoc test; *p < 0.05, **p < 0.01 (Table S2). (D) Expression of cell marker genes in indicated organoids determined by RT‐qPCR. For RT‐qPCR analysis, Ct values of target genes in organoids derived from SAMP8 mice (3 and 10 months) were normalized to the reference gene (Gapdh) to obtain ΔCt values. Age‐matched SAMR1 organoids served as the control group, and ΔΔCt values were calculated relative to the mean ΔCt of controls. Relative gene expression (or fold change) was determined using the 2^−ΔΔCt^ method. Statistical analysis was performed on fold‐change values from organoids derived from individual animals. (*n* = 5 animals per genotype, per sex, per time point). For visualization and interpretation, fold changes were log2‐transformed and displayed in heatmaps (downregulation in blue and upregulation in red). Immunohistochemistry and immunofluorescence analyses of the indicated organoids for mucin (E), chromogranin A (Chga, in green, indicated by white arrows) (F), and proliferation marker Ki67 (G) expression (*n* = 4 animals for staining, per genotype, per sex, per time point; ten organoids/image field for each animal were analyzed; pooled single measurements are shown). Scale bar: 100 μm. Values are presented as mean ± SEM. Statistical analysis was performed by one‐way ANOVA with Sidak's post‐test; *p < 0.05, **p < 0.01, ***p < 0.001 (Table S2). Abbreviations: M, month; S1, SAMR1; S8, SAMP8.

As an altered crypt depth was observed in the gut tissue of aged mice (see Figure 1A), we normalized the growth capacity to crypt number when investigating organoid growth capacity to prevent differences in preparation from affecting the outcome of growth capacity measurement (Figure 2A–C). Nevertheless, colonic organoid forming efficiency decreased significantly with age in both male and female SAMP8 mice and male SAMR1 mice (Figure 2A–C, Table S2). To further elucidate the underlying mechanism of this impaired growth, the expression of epithelial cell marker genes was quantified via RT‐qPCR (Figure 2D). When comparing values obtained for organoids of SAMR1 and SAMP8 mice, the expression of the stem cell marker *Lgr5* (Leucine‐rich repeat containing G protein‐coupled receptor) and of the proliferation marker *Ki67* was found to be reduced, while the expression of markers for goblet cells (*Muc2*, mucin 2) and enterocytes (*Krt20*, keratin 20) was found to be increased. *Chga*, a marker for EECs, was however decreased in organoids from SAMP8 mice. Interestingly, similar to the impairment in the growth capacity, organoids from SAMP8 male mice appeared to show changes in the expression of cell marker genes at a young age. In females these effects only became apparent with increasing age. The observed alterations in gene expression already indicated that, similar to the age‐related changes observed in the colon tissue, an increase in goblet cells might also be found in the organoids from aged mice. To analyze this further, the same markers used for colonic tissue were applied in immunohistochemistry experiments on organoids (Figure 2E–G). An increase in goblet cells was observed with age in organoids derived from SAMP8 mice and aged SAMP8 mice compared to SAMR1 mice (Figure 2E). Additionally, enteroendocrine marker expression and proliferation rates were decreased in organoids derived from both young and aged SAMP8 mice compared to SAMR1 mice (Figure 2F,G). These data suggest that certain age‐related features of the gut tissue are also preserved in colonic organoids, making them a valuable model for studying gut aging in vitro.

### 3.2 Proteomic Profile of Gut‐Derived Organoids

To gain a more detailed understanding of how aging manifests in the colonic organoids during healthy and accelerated aging, a proteomics analysis was performed. The proteomic profiles of organoids derived from young and aged SAMP8 and SAMR1 mice were compared. Firstly, we assessed the protein levels of several key markers that had previously been investigated using RT‐qPCR and immunofluorescence, such as Muc2, Krt20, and Chga (see Figure 2D–G). Unfortunately, however, these markers did not change significantly as expected. This could be due to unforeseen batch effects in the four proteomic processing steps per condition.

**FIGURE 3:**
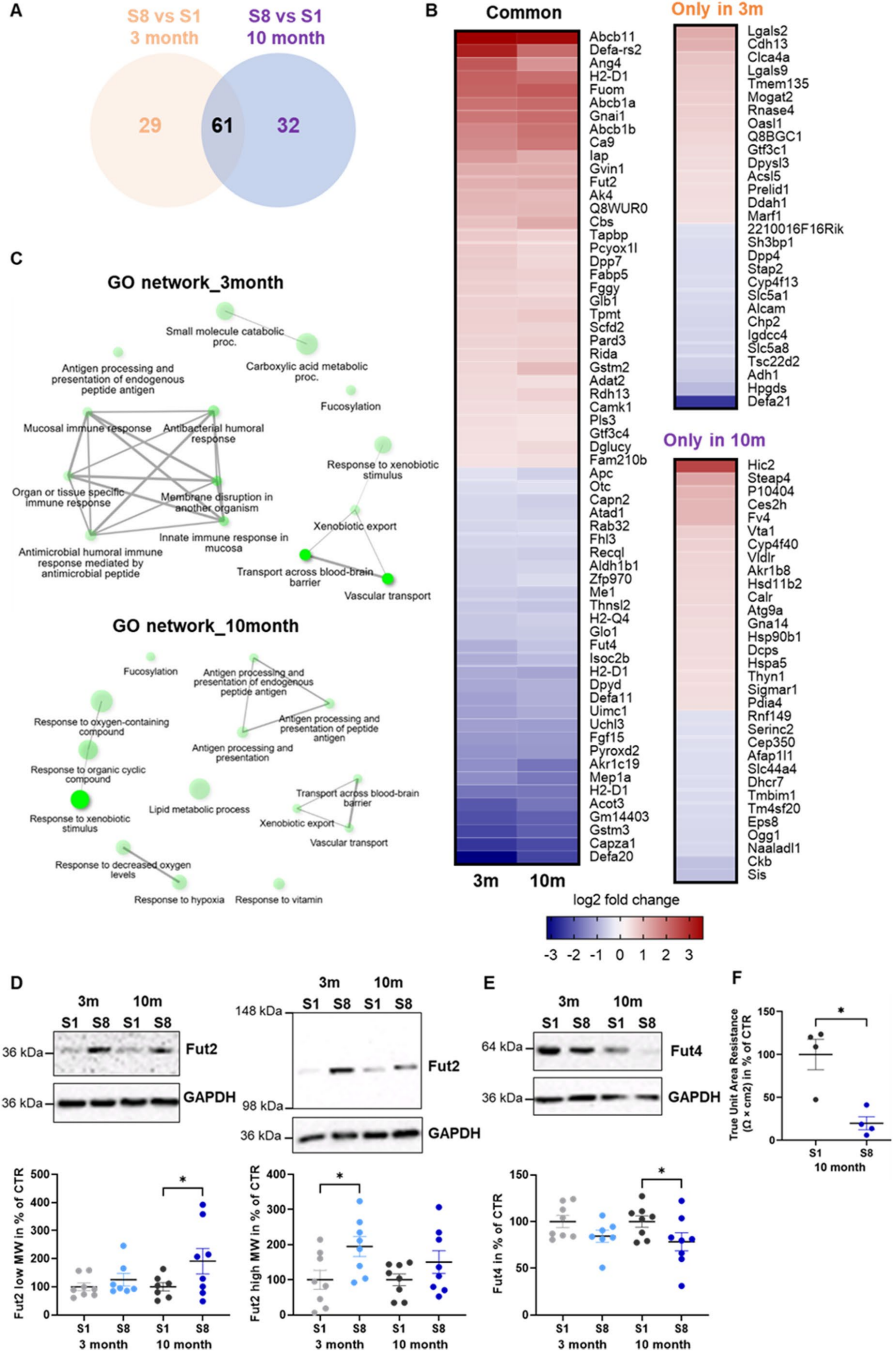
Proteomic profile of gut epithelium‐derived colonic organoids. (A) Venn diagram illustrating the unique and shared differentially abundant proteins (DAPs) in organoid samples derived from SAMP8 compared to SAMR1 mice at 3 and 10 months of age (orange, DAPs expressed only in SAMP8 versus SAMR1 at 3 months; purple, DAPs expressed only in SAMP8 versus SAMR1 at 10 months; black, DAPs expressed in common). (B) Heatmap showing the changes in protein expression level of DAPs (log2 fold change) in organoid samples derived from SAMP8 compared to SAMR1 mice at 3 months only, 10 months only, and at both ages. (C) Gene Ontology enrichment analysis (top 15 terms involved in biological processes) among all DAPs in organoid samples derived from SAMP8 compared to SAMR1 mice at 3 months and at 10 months of age, using ShinyGO 0.80 (http://ge‐lab.org/go/) with background correction. The interaction plot shows the relationship between enriched pathways. Two pathways (nodes) are connected if they share 20% (default) or more genes. Darker nodes are more significantly enriched gene sets. Bigger nodes represent larger gene sets. Thicker edges represent more overlapped genes. *n* = 4 per genotype, per sex, per time point (Table S2). A minimum fold change of 1.30‐fold and a *p*‐value threshold of 0.05 were used. (D) Western blot analysis of Fut2 expression (protein bands observed at low and high molecular weights (MW)), with GAPDH serving as a normalization control, in colonic organoids derived from young and aged SAMR1 and SAMP8 mice. A representative Western blot of Fut2 detected in colonic organoid samples derived from female SAMR1 and SAMP8 mice is shown. (E) Western blot analysis of Fut4 expression, with GAPDH serving as a normalization control, in colonic organoids derived from young and aged SAMR1 and SAMP8 mice. A representative Western blot of Fut4 detected in colonic organoid samples derived from male SAMR1 and SAMP8 mice is shown. For Western blot quantitation, *n* = 8 (4 females and 4 males) animals per genotype and per time point were used. Values are presented as mean ± SEM. Statistical analysis was performed using one‐way ANOVA followed by Fisher's LSD post hoc test; *p < 0.05 (Table S2). (F) Transepithelial electrical resistance (TEER) analysis of cellular monolayers derived from colonic organoids from aged SAMP8 mice compared with those from aged SAMR1 mice, expressed as percentage (*n* = 4 female animals per genotype). Statistical significance between the two groups was assessed using an unpaired two‐tailed Welch's *t*‐test; *p < 0.05 (Table S2). Abbreviations: CTR, control; m, month; MW, molecular weight; S1, SAMR1; S8, SAMP8.

Nevertheless, we identified around 90 differentially abundant proteins (DAPs), when comparing organoids derived from SAMP8 and SAMR1 mice in each age group (Figure 3A). Around 60% of these DAPs overlapped and displayed similar trends across the two age groups (Figure 3A,B). We also performed Gene Ontology (GO) enrichment analysis using ShinyGO (Figure 3C, Table S3). Specifically, our data revealed an abundance of proteins that are expressed in the intestine, including a subset of antimicrobial peptides (e.g., alpha‐defensins: Defa11, Defa‐rs2, Defa20, and Defa21; Figure 3B,C). Beta‐defensins are abundantly produced by colonic epithelial cells, while alpha‐defensins are mainly secreted by Paneth cells of the small intestine and neutrophils. However, in a cancerous environment, also colonic alpha‐defensins have been described (Qiao et al. 2021), thus, the accelerated aging might here also contribute to aberrant expression. In addition, members of the ATP‐binding cassette (ABC) transporter family (including Abcb1a, Abcb1b, and Abcb11) also displayed higher abundance in organoids of SAMP8 than in SAMR1 at both ages. These proteins are involved in xenobiotic export (Figure 3B,C), are situated in the apical membrane of intestinal epithelial cells and play a crucial role in determining the distribution of many orally administered drugs (Mizutani et al. 2012). Among the DAPs that we identified, we found increased levels of proteins related to the immune response to pathogens, including antimicrobial peptides (e.g., Defa‐rs2 [alpha‐defensin‐related sequence 2], Ang4 [angiogenin‐4], in both age groups), major histocompatibility complex (MHC) proteins (e.g., H2‐D1, in both age groups) and galectins (e.g., Lgals2 and Lgals9, in the young group only). Interestingly, the levels of inflammation‐related proteins such as Gvin1 (interferon‐induced very large GTPase 1) and Iap (IgE‐binding protein), were increased in both age groups (Figure 3B,C). Fut2 (galactoside alpha‐(1,2)‐fucosyltransferase 2), an important protein involved in fucosylation in response to intestinal inflammation and infection, was also found to be upregulated in both age groups (Figure 3B). This is consistent with a previous finding (Z. H. Wang et al. 2024), which showed that Fut2 is upregulated in older individuals. Furthermore, distinct DAPs were identified in organoids from aged SAMP8 mice compared to those from aged SAMR1 mice. Notably, Hic2 (Hypermethylated in cancer 2) was strongly upregulated, while Ckb (Creatine kinase B‐type), which plays a protective role in active mucosal inflammation (Gebert et al. 2020), was downregulated (Figure 3B). Further analysis of the interaction network revealed a cluster of proteins associated with hypoxic stress, which typically arises when the demand for cellular oxygen exceeds its supply. This phenomenon is commonly observed in tissues subjected to infection and inflammation (Zeitouni et al. 2016). Furthermore, proteins associated with lipid metabolism were identified (Figure 3C).

To evaluate the proteomics findings, Fut2 and Fut4 (alpha‐(1,3)‐fucosyltransferase 4) were exemplarily selected for validation using Western blotting. They belong to the fucosyltransferase family, which plays a key role in regulating cell–cell interactions through glycan modification, thereby influencing microbiota composition, epithelial barrier function, and inflammatory responses (Ghirardello et al. 2025). Notably, these proteins showed opposing regulation in the proteomics dataset (Figure 3B), enabling us to assess whether the observed changes reflect a coordinated shift in epithelial fucosylation rather than stochastic variation. Fut2 is reported to have a molecular weight (MW) of about 37 kDa (Z. Wang et al. 2023; Zhang et al. 2025). However, besides a respective faint band around 37 kDa, we observed an additional band at a higher MW (about 120 kDa). The latter band was nearly absent in heart (a tissue having only trace amounts of Fut2), while being present in colon and being regulated by fucose treatment in colonic organoid cultures (Z. Wang et al. 2023) (see Figure S5A). Both bands were quantified independently to investigate age‐related expression of the enzyme. We observed that levels of Fut2 with low MW were significantly increased in organoids derived from aged SAMP8 as compared to SAMR1‐derived organoids (Figure 3D, left panel; Figure S5B). Additionally, the protein showing high MW was increased in organoids derived from young SAMP8 (Figure 3D, right panel; Figure S5B) compared to age‐matched organoids derived from SAMR1 controls. For Fut4, different molecular weights have been reported (e.g., > 59 kDa in ovarian carcinoma cells (Escrevente et al. 2006)). In a pre‐test, we detected a band at around 60 kDa that was absent in heart (negative control), present in bone marrow (positive control), and reduced following fucose treatment of colonic organoids (Figure S5C). Fut4 protein levels were significantly higher in organoids from aged SAMP8 mice compared with age‐matched SAMR1 controls (Figures 3E and S5D). Overall, these results confirm the proteomics findings for the selected targets by Western blotting.

Our proteomic data showed an increased abundance of antimicrobial peptides, along with protein–protein interaction networks associated with immune responses and inflammatory conditions, indicating alterations in pathways related to epithelial function. To functionally assess these changes, we evaluated epithelial barrier integrity using transepithelial electrical resistance (TEER). In line with these pathway changes, TEER measurements demonstrated a significant reduction of resistance in organoids derived from aged SAMP8 mice compared with age‐matched SAMR1 controls (Figure 3F), indicating impaired epithelial barrier function. Together, these findings provide functional support for the proteomics‐derived signatures and suggest that aging‐associated alterations may compromise epithelial integrity.

### 3.3 A Certain Stability of the Observed Phenotypes Was Shown in Organoids at Different Passages

**FIGURE 4:**
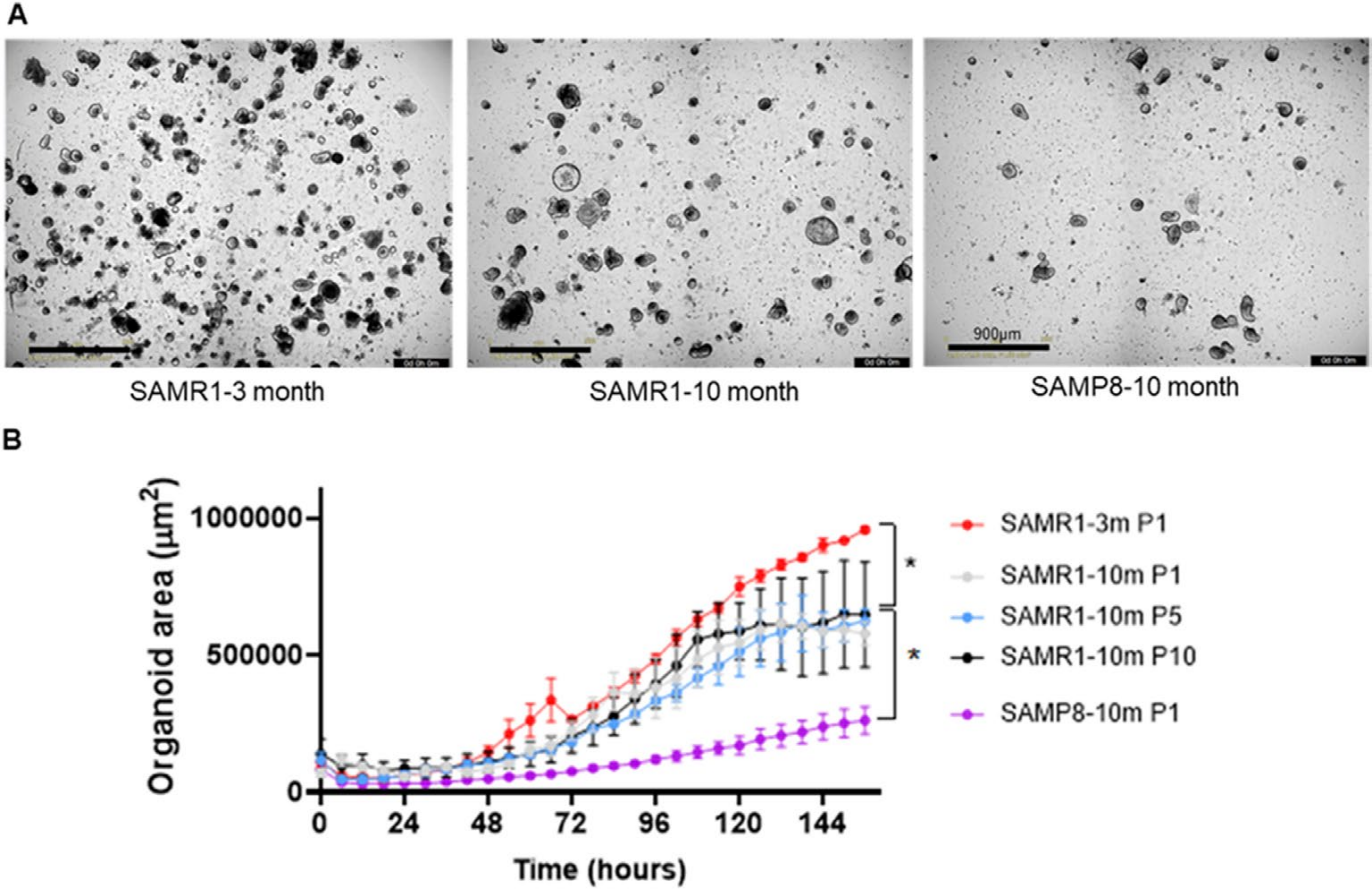
Analysis of growth potential of single‐clonal organoid cultures from SAMR1 mice after passaging. Growth capacity of single‐clonal organoid cultures derived from SAMR1 mice aged 3 and 10 months at different passage numbers (P1, P5, and P10), as well as from SAMP8 mice aged 10 months (*n* = 4 female animals), was investigated. (A) Representative images of organoids at the experimental endpoint (Day 7). Scale bar: 900 μm. (B) Organoid area curve over time (μm^2^). Values are presented as mean ± SEM. Statistical analysis using end values of incubation time was performed by one‐way ANOVA with Sidak's post‐test; *p < 0.05 (Table S2). Abbreviations: M, month; P, passage number.

Our study shows that certain age‐related features of gut tissue are also preserved in the colonic organoids and that accelerated aging can be modeled using these organoids. However, it is unclear whether these features are permanently present or whether the underlying epigenetic regulation is lost over time. In order to investigate this further, and to establish how long age‐related changes in the gut might be preserved in the gut‐derived organoid model, organoids were derived from aged control mice at different passages (P1, P5, and P10), as well as from young control mice and aged SAMP8 mice at passage 1 (P1). The growth properties of these organoids were then investigated using single‐clonal organoid culture regarding an Incucyte live cell microscope (Figure 4A,B). As in our previous observations, a comparison of passage 1 revealed that the stem cells of young control mice showed the most potent growth, while the stem cells of aged SAMP8 mice showed the least growth (Figure 4A,B). Organoids derived from old SAMR1 control mice showed an intermediate growth phenotype; however, passages 1, 5, and 10 of these organoid cultures were indistinguishable. This suggests that the age‐dependent organoid phenotypes—at least in terms of the growth capacity—are encoded intrinsically and do not change with prolonged culture. Therefore, they can be studied in vitro for at least 10 passages.

### 3.4 Age of the Gut Epithelium Has a Greater Impact on the ENS‐Organoid Co‐Culture System

The gut epithelium acts as a barrier between the microbiota and the underlying cell layers such as the ENS. Therefore, the interplay between these tissues and between them and the microbiota may be an important driving force in the aging process of the entire organ system and the organism. We therefore decided to first study the interplay using a co‐culture system incorporating aging in one or both of the interacting tissue components. A critical initial step was the identification of a culture medium that preserves the functionality of both epithelial and ENS compartments. Notably, the cENR medium, which was used for organoid culture, proved to be well suited for this purpose. It supported epithelial growth while maintaining ENS function, as evidenced by preserved AChE activity (Figure S6A,B) and a cell viability as well as caspase‐1 activity comparable to those observed under standard ENS culture conditions (Figure S6C,D).

**FIGURE 5:**
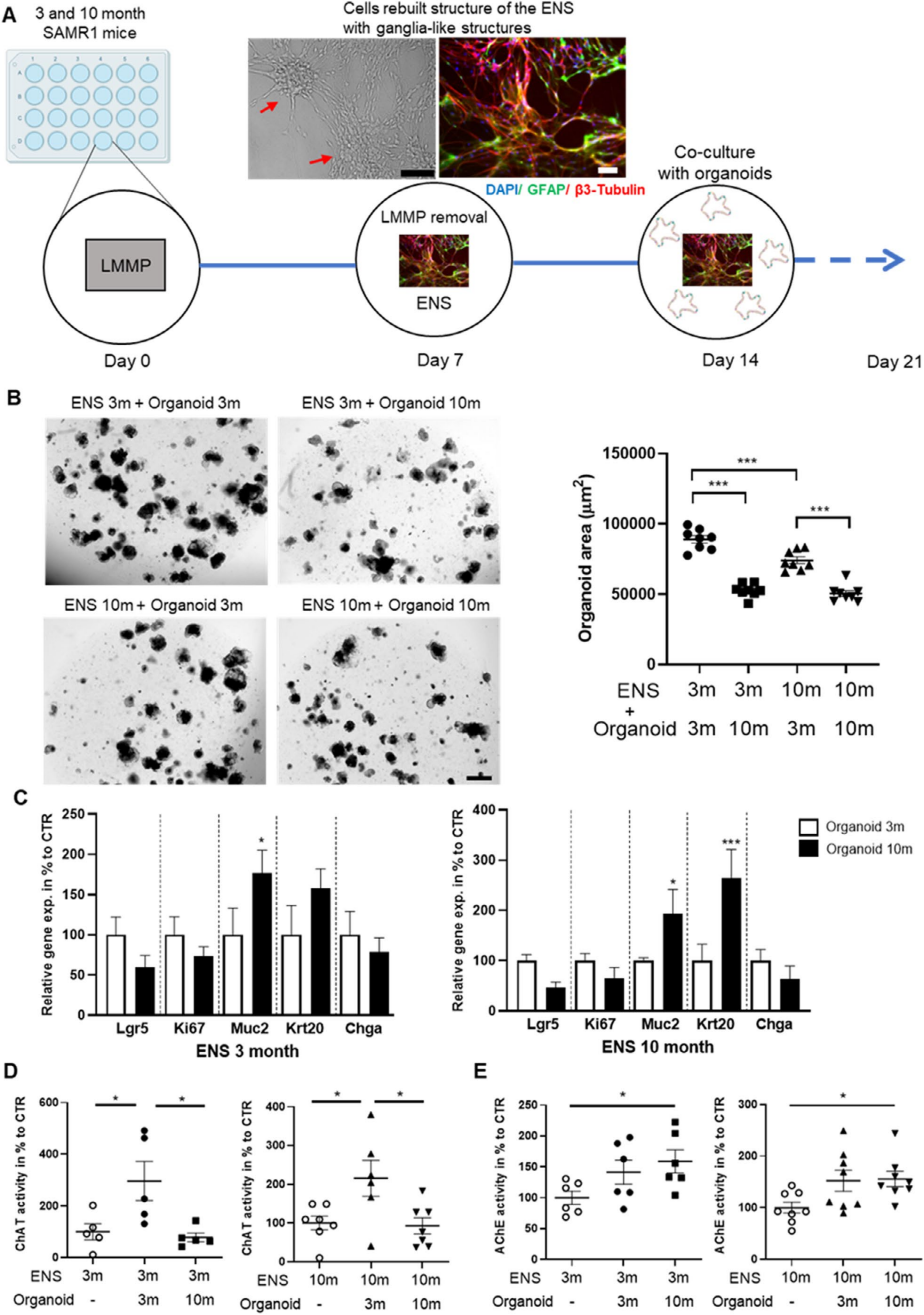
Mutual age‐dependent interrelationship of the epithelium and ENS in co‐culture. To investigate the effect of an aged ENS on epithelium and vice versa, the ENS was co‐cultured with colonic organoids as a model of gut epithelium. (A) The co‐culture experiment using female SAMR1 mice aged 3 and 10 months. The ENS cells were stained with DAPI (nucleus, in blue), β3‐tubulin (neuronal marker, in red), and GFAP (glial marker, in green). Ganglia‐like structures are indicated by red arrows. Scale bar: 100 μm. (B) Comparison of organoid total area in the ENS‐organoid co‐culture systems with ENS derived from young and aged mice (*n* = 5 animals per time point, Table S2). Scale bar: 200 μm. (C) Characterization of organoids in the co‐culture systems using RT‐qPCR (*n* = 5 animals per time point, Table S2). (D) Choline acetyltransferase activity of the ENS in the co‐culture system (*n* = 5–7 female animals per time point, Table S2). (E) Acetylcholinesterase activity of the ENS in the co‐culture system (*n* = 5–7 female animals per time point). Values are presented as mean ± SEM. Statistical analysis was performed by one‐way ANOVA with Sidak's post‐test; *p < 0.05, ***p < 0.001 (Table S2). Abbreviations: AChE, acetylcholinesterase; ChAT, choline acetyltransferase; CTR, control; ENS, enteric nervous system; GFAP, glial fibrillary acidic protein; LMMP, longitudinal muscle/myenteric plexus; m, month.

We cultured the ENS from young and aged female SAMR1 mice using a stamp‐like approach. Cells that migrated from the LMMP reconstructed the ENS architecture by forming ganglia‐like structures that stained positively for both neuronal and glial markers (Figure 5A). The ENS cells from young and aged mice were then co‐cultured with colonic organoids derived from young and aged SAMR1 animals for 7 days. The total organoid area was analyzed. Regardless of the age of the donor of the used ENS, organoids derived from older mice exhibited a decreased growth capacity compared to those obtained from young animals (Figure 5B, growth capacity nearly halved). Introducing an aged ENS to organoids derived from young mice resulted in a slight but significant further reduction in organoid growth compared to organoids co‐incubated with same‐age ENS. In organoids from aged individuals, the aged ENS did not decrease growth further. When analyzing cellular composition by the aforementioned marker gene expression (Figure 5C), a minor effect of the aged ENS could also be detected. For example, organoids derived from young and aged animals differed in *Muc2* expression by a factor of 1.7, regardless of whether they were exposed to ENS derived from young or aged animals. This finding confirms the observation that goblet cell numbers increase with age. All the other markers tested revealed a non‐significant decrease when comparing co‐cultures with organoids from young or aged animals. The only marker for which aged ENS led to changes compared to co‐culture with young ENS was *Krt20*, which is a marker for enterocytes. Its expression increased by 150% in the presence of aged ENS, whereas organoids in co‐culture with young ENS showed an increase of only 50% compared to control.

Finally, we were interested to see how the function of the ENS is impacted by aged epithelium (organoids). Therefore, we selected the following key enzymes in cholinergic signaling as they play an important role in gut innervation: ChAT and AChE. In the co‐culture experiments, ChAT activity significantly increased in co‐cultures of organoids from young donors and both ages of ENS compared to the enzyme activity of ENS cultivated alone (Figure 5D). However, whenever organoids from aged donors were introduced, ChAT activity within the ENS decreased drastically in both young and aged ENS (Figure 5D). Co‐culture with aged organoids induced AChE activity in both young and aged ENS (Figure 5E). When organoids derived from aged donors were introduced, a slight further increase was observed in comparison to co‐culture with organoids derived from young mice; however, this did not reach statistical significance. Overall, these results suggest that the epithelium itself impacts on the functionality of the ENS, and that it exerts a comparably strong age‐dependent effect at least on some parameters.

### 3.5 Impact of the Age of FW Donors on Colonic Organoid Characteristics

**FIGURE 6:**
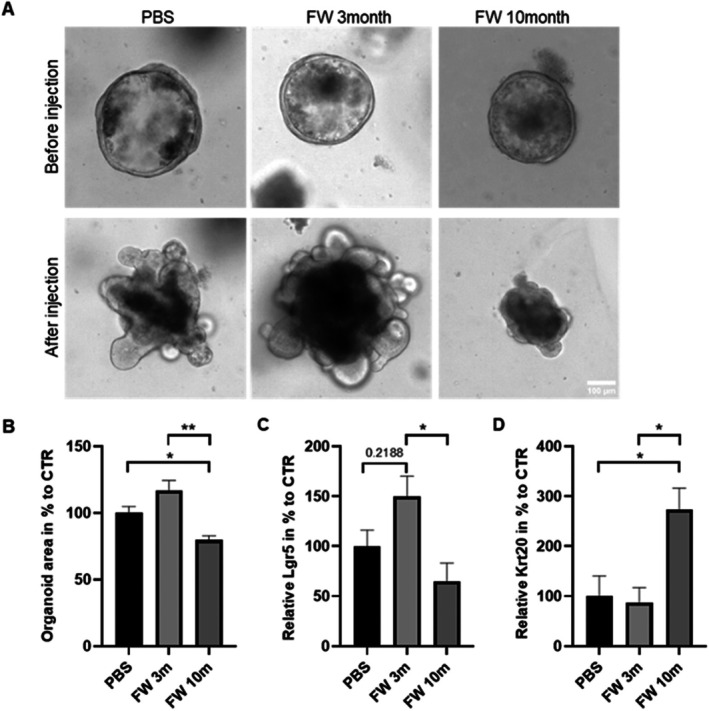
Impact of the age of fecal water donors on colonic organoid characteristics. Feces from young and aged female SAMR1 mice (*n* = 5 animals per age) were collected and pooled for each age group. Colonic organoids of young SAMR1 mice were injected with PBS as control and FW derived from young and aged SAMR1 mice (*n* = 5 animals per time point, Table S2). (A) Representative images of organoids before (at Day 4) and after PBS or FW injection from young and aged donors (at Day 7). Scale bar: 100 μm. (B) Organoid total area in percentage normalized to PBS‐injected organoid as control. (C) RT‐qPCR data of organoids after FW injection from young and aged donors, showing expression of the stem cell marker Lgr5 normalized to PBS‐injected organoids. (D) RT‐qPCR data of organoids after FW injection from young and aged donors, showing expression of the enterocyte marker Krt20 normalized to PBS‐injected organoids. Values are presented as mean ± SEM. Statistical analysis was performed by one‐way ANOVA with Sidak's post‐test; *p < 0.05, **p < 0.01 (Table S2). Abbreviations: CTR, control; FW, fecal water; m, month.

In addition to interacting with other cell types in the host, the gut epithelium is exposed to external environmental factors such as the microbiota. FW reflects the metabolome of the commensal microbiota, while it, aside, may contain material from shed host cells or secreted compounds derived by host cells. We therefore injected FW derived from the feces of young and aged female SAMR1 mice into organoids isolated from young female SAMR1 mice. Using the same strain and sex for FW donors and organoid recipients should at least eliminate a vast amount of strain‐ and sex‐specific factors elicited by a differing host type and allow investigation of the microbiota effect. The success of the microinjection was demonstrated by injecting propidium iodide (Figure S1). As PBS was used as the solvent for homogenizing the feces, PBS injection was performed as a sham injection to indicate effects caused by the mechanical disruption due to the needle insertion. Injecting FW from aged mice into organoids derived from young mice resulted in a significant reduction in organoid size compared to organoids injected with FW from young mice (Figure 6A,B). This morphological change was accompanied by a decrease in stem cell marker (*Lgr5*) expression, as determined by RT‐qPCR (Figure 6C), indicating reduced stemness. In parallel, the expression of the enterocyte differentiation marker *Krt20* was increased (Figure 6D). These phenotypes are similar to those observed in organoid monocultures derived from aged animals compared to those from young animals.

## 4 Discussion

This study identifies the intestinal epithelium as a key driver of colonic aging, demonstrating that age‐related structural and cellular phenotypes are stably preserved in epithelial‐derived organoids. The persistence of these features in vitro suggests that epithelial aging is largely cell‐intrinsic, while remaining highly responsive to extrinsic cues such as microbiota‐derived metabolites and, to a lesser extent, ENS signaling.

### 4.1 Epithelial Aging Limits Regenerative Capacity

SAMP8 mice displayed increased colonic crypt depth and reduced epithelial proliferation, consistent with chronic inflammation (Toritani et al. 2022) and impaired tissue renewal. Such an increase in crypt depth was, for example, also observed in ileum in C57BL/6 mice that were 18–26 months old compared to mice at 2–5 months old (Nalapareddy et al. 2017). Although crypt architecture cannot be directly assessed in organoids due to their spherical character, reduced organoid forming efficiency and proliferation provided functional correlates of stem cell dysfunction. These defects were evident in both young and aged SAMP8 mice relative to SAMR1 controls, suggesting premature exhaustion of the stem cell compartment. This decline can be associated with an increase in cell cycle arrest through TGF‐β‐Smad3 signaling as has been shown for colonic organoids obtained from C57BL/6 mice that were 20 months old compared to mice at 6–10 weeks old (Jo et al. 2023).

Reduced Ki67 labelling in colonic tissue and organoids is consistent with previous reports in murine and human intestines, supporting the idea that a decline in epithelial regenerative capacity is a hallmark of aging (Nalapareddy et al. 2021; Tomasetti et al. 2019). For instance, one study of human tissue found that the rate of cell proliferation in colonic tissue using Ki67 labeling was significantly lower in an older cohort (80–89 years, *n* = 13; reduction by 41%) than in the younger cohort (20–29 years, *n* = 13) (Tomasetti et al. 2019).

### 4.2 Aging Signatures Are Stable in Colonic Organoids for Several Passages

Age‐related differences in growth and proliferative behavior were maintained through at least ten organoid passages, indicating the stable propagation of aging phenotypes. This persistence is likely mediated by conserved epigenetic features, including DNA methylation patterns that encode an epithelial aging signature (Lewis et al. 2020). Analysis of human intestinal epithelial organoids derived from various cohorts (*n* = 131, including both pediatric and adult samples) revealed that passage number—from early stages (passage 1, ~7–10 days) to later stages (passages 11–16, ~4–10 months)—accounted for approximately 10% of the variability in DNA methylation patterns (Edgar et al. 2022). A validation subset from this study (*n* = 51) revealed that a portion of CpG (cytosine‐phosphate‐guanine dinucleotide) sites exhibited passage‐related methylation shifts: 5.2% showed heteroskedasticity, 2.2% underwent hypomethylation, and 0.8% showed hypermethylation. Furthermore, a separate cohort from the same study (*n* = 42, pediatric) indicated that prolonged culture (2 vs. 12 passages) did not affect organoid morphology, growth, epithelial barrier integrity, or the expression of specific markers such as *LGR5* and *MUC2*, and enterocyte markers such as fatty acid binding proteins (*FABP1* and *FABP6*) (Edgar et al. 2022). Thus, while prolonged culture may introduce limited epigenetic drift, our findings support the suitability of the use of intestinal organoids from mice as a robust model for mechanistic studies of epithelial aging.

### 4.3 Aging Reshapes Epithelial Lineage Allocation

Aging was associated with significant changes in the composition of epithelial cells. The abundance of EEC was reduced in both tissue and organoids derived from SAMP8 mice, which could potentially impair the hormone‐mediated regulation of metabolism and epithelial homeostasis. Consistent with this interpretation, proteomic analyses revealed age‐related alterations in lipid metabolism pathways, suggesting functional consequences of diminished EEC signaling. However, the literature on age‐related changes in these cells is contradictory. One study on NMRI (Naval Medical Research Institute) mice showed that GIP (glucose‐dependent insulinotropic polypeptide)‐ and SST (somatostatin)‐immunoreactive EECs decreased in mice aged 12 and 24 months as compared to younger individuals, while secretin‐immunoreactive EECs increased in mice aged 24 months compared to mice aged 3 months (Sandstrom and El‐Salhy 2000). However, another study using mice with fluorescently labeled Lgr5 showed no change in number of EECs of mice aged 17–24 months compared to young mice aged 3–4 months (Mihaylova et al. 2018). A decline in EECs can impair the secretion of essential hormones (e.g., GLP‐1) (Gribble and Reimann 2019), which can ultimately impair the colon's function in digestion and metabolism. For instance, reduced GLP‐1 secretion could disrupt glucose homeostasis, accelerate gastric emptying, and alter satiety signals, potentially resulting in hyperglycemia, as observed in aged SAMP8 mice (Cuesta et al. 2013). GLP‐1 also regulates lipid metabolism, as demonstrated by the impaired secretion of GLP‐1 from EECs in V1aR/V1bR double‐deficient mice (lacking vasopressin receptors V1a and V1b), which exhibited abnormal lipid accumulation (Harada et al. 2025). Our proteomic analysis revealed DAPs in comparison of organoids derived from aged SAMP8 compared to SAMR1 mice that were associated with lipid metabolism. These DAPs may be linked to the age‐related decline in EEC hormone secretion.

In contrast to EECs, the proportion of goblet cells increased with age and in the accelerated senescence phenotype. Using single‐cell RNA sequencing, an increase of around 20% in the number of goblet cells was observed in the colon of C57BL/6 mice (aged 89–117 weeks compared to 8–18 weeks) during aging (Sirvinskas et al. 2022). Furthermore, alcian blue staining revealed that a 40% increase in the average number of goblet cells in the small intestines of aged C57BL/6 mice (18–22 months vs. 2–4 months) was shown (Nalapareddy et al. 2017). This shift towards secretory differentiation likely reflects declining intestinal stem cell potency and attenuated Wnt signaling, favoring terminal differentiation. Increased goblet cell abundance may represent a compensatory response to epithelial stress, inflammation, or microbiota dysbiosis by enhancing mucus‐mediated barrier protection. This may be related to the increased demand for mucus to protect the intestinal lining from age‐related damage and inflammation (Zheng et al. 2022), consistent with proteomic changes in antimicrobial peptides and MHC proteins in organoids derived from SAMP8 mice. In line with this, Fut2 upregulation in SAMP8 organoids indicates altered epithelial glycosylation, as FUT2‐mediated fucosylation of mucins and epithelial glycans modulates mucus properties and host–microbiota interactions (Pickard et al. 2014). However, these changes were accompanied by decreased TEER, indicating impaired barrier integrity. Thus, despite increased goblet cell abundance and potential enhancement of mucus‐related pathways, epithelial function is compromised.

### 4.4 Bidirectional Epithelial–ENS Crosstalk Is Altered by Aging

We have developed a simplified and efficient approach to isolating and expanding ENS cells, which enables the systematic investigation of epithelial–ENS interactions during aging. Traditional methods require extensive preparation as well as a complex enzyme digestion process, which can result in the loss of cell*–*cell contacts and cell death (Smith et al. 2013). Previous studies have already demonstrated that aging obstructs intestinal epithelial regeneration, resulting in leaky intestinal barriers (Branca et al. 2019). In humans, the loss of this regenerative capacity in the epithelium has been attributed to age‐related changes of the stem‐cell niche (Pentinmikko and Katajisto 2020). Similarly, isolated studies with intestinal organoids have demonstrated that the age of the donor impacts the capacity for organoid growth in both mice and humans (Nalapareddy et al. 2017; Pentinmikko et al. 2019). However, the mechanisms and extent to which the ENS affects epithelial growth during aging remain grossly unclear (Pentinmikko and Katajisto 2020).

In co‐culture, epithelial age, but not ENS age, was the primary determinant of reduced organoid growth. This highlights the dominant role of epithelial‐intrinsic aging in limiting regenerative capacity. Notably, epithelial age also modulated ENS cholinergic signaling. Young organoids increased ChAT activity in ENS cells, probably through epithelial‐derived acetylcholine signaling from tuft cells or other non‐neuronal sources. In contrast, aged organoids selectively increased ENS AChE activity, suggesting that epithelial aging alters the cholinergic balance and may suppress ENS function. These findings emphasize the previously underappreciated role of epithelial aging in shaping ENS neurotransmitter dynamics.

It should be noted that, in addition to being found in neurons, ChAT is also expressed in the intestinal epithelium, primarily through tuft cells (Middelhoff et al. 2020). The cellular origin of tuft cells is associated with Lgr5‐expressing ISCs, which were also implicated in the cultivation of colonic organoids used in this study (Sato et al. 2009). Similarly, studies have demonstrated that crypt‐villus organoids have functioning non‐neuronal cholinergic signaling systems with AChE expression (Takahashi et al. 2014). However, as the neuronal tissue was removed separately from the co‐cultures for measuring the components of the cholinergic pathway, it can be concluded that enzymes derived from the epithelium (organoids) did not contribute to the observations described here.

### 4.5 Microbiota‐Derived Metabolites Recapitulate Epithelial Aging Phenotypes

The injection of FW from aged animals into organoids induced phenotypes resembling those of aged epithelium, implicating microbiota‐derived metabolites as key extrinsic modulators of epithelial aging. It is known that age‐related reductions in short‐chain fatty acids (SCFAs) (Hirabayashi et al. 2020) and serotonin 5‐HT (Kwon et al. 2019) impair epithelial integrity and stemness and likely contribute to the observed decline in regenerative capacity. For instance, the loss of butyrate‐producing bacteria with age and the resulting reduction in butyrate levels contribute to decreased 5‐HT levels in the elderly (Ghare et al. 2022). A 70% decrease in fecal SCFAs was observed in aged mice (Hirabayashi et al. 2020). Furthermore, transfer of aged microbiota into young mice increases intestinal tumor necrosis factor (TNF) levels and markers of epithelial barrier disruption (intestinal fatty acid‐binding protein I‐FBP, lipopolysaccharide‐binding protein LBP) (Parker et al. 2022), implicating microbiota‐driven inflammation in epithelial dysfunction. These observations suggest that aging‐associated alterations in microbial metabolism may substantially reshape the luminal environment sensed by the intestinal epithelium. In this context, we show that FW derived from aged mice suppresses ISC activity in a young epithelium, evidenced by reduced *Lgr5* expression and impaired organoid growth. This aligns with prior work demonstrating that ISCs from aged animals exhibit intrinsically diminished regenerative capacity, associated with a set of transcription factors, including upregulation of *Nfe2l2* (nuclear factor erythroid 2‐related factor 2) and downregulation of *Irf1* (interferon regulatory factor 1), *Fosb* (Fos B proto‐oncogene, AP‐1 transcription factor subunit), and *Egr1* (early growth response 1) during aging, which are key driving the transition from a young to an aged ISC state, as well as epigenetic remodeling (including DNA methylation) of stemness‐associated programs (Nefzger et al. 2022). These data support a model in which epithelial aging emerges from the convergence of intrinsic epigenetic programs and extrinsic microbial signals.

Regardless of the usability of our co‐culture model and the related findings, we have to address some relevant limitations to our study. Firstly, we used murine cells for our investigations. While some general aspects may be comparable between mouse and human, this might be relevant in regard to clinical translation perspectives. One example is the family of defensins, which highly differs between species due to copy number variations and SNPs (van Dijk et al. 2023) and thus findings might not be directly transferred. A second limitation is that we did not define the exact passage number by which organoids might lose their respective phenotype. However, ten passages already allow a certain time corridor for investigations. Next, the current study primarily infers ENS maturation from morphology and limited marker expression; more comprehensive characterization of the migrating population—including quantitative neuronal and glial markers and functional subtype analysis—is needed to strengthen these conclusions. Another limitation is the FW injection experiments were only conducted in organoids derived from female tissue donors. As, for example, the analysis of organoid growth capacity and cell markers revealed sex‐specific differences for the organoids, such differences might also occur in the reactivity towards FW components. This needs further investigation in future as well as the fact that the FW preparation was based on a water extraction: hydrophobic compounds thus might not have been addressed by these experiments. Further studies using additional aging models (e.g., SAMP8 mice) are warranted to gain a more comprehensive understanding. Finally, a limitation is the modest number of biological replicates (*n* = 3–8 animals per experiment), which is common in similar studies; nevertheless, the consistency of effects across experiments supports the robustness of the findings, while generalizability should be interpreted with appropriate caution.

## 5 Conclusion

Together, our findings position the intestinal epithelium as a central integrator of aging signals arising from intrinsic cellular programs, microbial metabolism, and ENS crosstalk. The consistent transfer of aging phenotypes to organoids establishes this system as a robust platform for investigating mechanisms of gastrointestinal aging and for evaluating interventions that target epithelial rejuvenation, microbial metabolites, or neuroepithelial signaling pathways in future investigations.

## Author Contributions

T.T.N. conceptualized the analysis, analyzed data, constructed illustrations, and wrote the manuscript. D.S., K.F., and M.A. performed some experiments and analyzed the data. J.‐X.C. supervised proteomics experiments and its data analysis. M.D. and F.K. performed proteomics data analysis. J.V. assisted in the establishment of organoid culture. J.P. and S.S. discussed, reviewed, and revised the manuscript. O.T. and P.B. carefully reviewed and revised the manuscript. K.E. conceptualized the study and analysis, reviewed and revised the manuscript. All authors have read and agreed to the published version of this manuscript.

## Funding

This work was supported by the German Academic Exchange Service (DAAD) Research Internships in Science and Engineering (RISE) Program 2024 (Funder DOI: 10.13039/501100001655). The Orbitrap Astral system was supported by the Deutsche Forschungsgemeinschaft (DFG, German Research Foundation) (Funder DOI: 10.13039/501100001659).

## Conflicts of Interest

The authors declare no conflicts of interest.

## Supporting information


**Figure S1:** Representative microinjection of propidium iodide dye into organoid. Images of organoids are shown in bright‐field (upper panel) or fluorescence (middle panel) or merge (lower panel). Images taken before and after (0, 1‐ or 3‐days) microinjection of propidium iodide dye with Nanoject III. Increasing thickness of organoid lumen indicated cell regeneration upon injection. Scale bar: 50 μm.
**Figure S2:** Reproducibility scatterplots for organoids derived from young SAMR1 and SAMP8. Scatterplot matrices depicting high reproducibility between replicates of the same batch (replicates 1 & 2 or replicates 3 & 4) based on log10‐transformed, normalized, and corrected protein intensities. Data are shown for organoids derived from young male (A) and female (B) SAMR1, and male (C) and female (D) SAMP8 mice. A batch effect is observed between the two batches of samples (replicates 1 & 2 vs. replicates 3 & 4). Abbreviations: F, female; M, male.
**Figure S3:** Reproducibility scatterplots for organoids derived from aged SAMR1 and SAMP8.Scatterplot matrices depicting high reproducibility between replicates of the same batch (replicates 1 & 2 or replicates 3 & 4) based on log10‐transformed, normalized, and corrected protein intensities. Data are shown for organoids derived from aged male (A) and female (B) SAMR1, and male (C) and female (D) SAMP8 mice. A batch effect is observed between the two batches of samples (replicates 1 & 2 vs. replicates 3 & 4). Abbreviations: F, female; M, male.
**Figure S4:** Principal component analysis plot of replicates for proteomics analysis. Principal component analysis of the 500 most variable proteins based on intensities following normalization by median‐centering. A batch effect is observed between the two batches of replicates. Abbreviation: PC, principal component.
**Figure S5:** Validation of antibodies against Fut2 and Fut4 for Western blot analysis.
To validate the antibodies against Fut2 and Fut4 used for Western blotting, murine tissues with known expression status, as well as colonic organoids treated with fucose (substrate of either enzyme), were analyzed. (A) Western blot analysis of Fut2 in murine heart (negative control), colon (positive control), and colonic organoids derived from young female SAMR1 mice without (−) and with (+) fucose treatment. (B) Representative Western blot of Fut2 in colonic organoids derived from young and aged male SAMR1 and SAMP8 mice, related to Figure 5D. (C) Western blot analysis of Fut4 in murine heart (negative control), bone marrow (positive control), and colonic organoids derived from young female SAMR1 mice without (−) and with (+) fucose treatment. (D) Representative Western blot of Fut4 in colonic organoids derived from young and aged female SAMR1 and SAMP8 mice, related to Figure 5E. GAPDH served as a loading control. Abbreviations: −, without; +, with; BM, bone marrow; C, colon; CO, colonic organoid; F, fucose; H, heart; m, month; S1, SAMR1; S8, SAMP8.
**Figure S6:** Comparison of ENS and organoid culture medium on ENS growth capacity and activity.
To compare the impact of the ENS and the organoid culture medium on ENS growth capacity and activity, LMMPs were cultured in standard ENS growth medium (ENS medium) and in cENR medium, which was used for organoid culture as well as for organoid‐ENS co‐culture. For this purpose, samples were isolated from young and aged female SAMR1 mice (*n* = 3 animals per age group, with *n* = 4 technical replicates per animal). (A) Images taken after 7 days of LMMP culture in ENS and cENR medium. Cells grew and migrated from LMMP tissue derived from animals at both ages in both media (indicated by arrowheads). Scale bar: 200 μm. (B) AChE activity of the ENS in both culture media. (C) Images taken after 14 days of LMMP culture in ENS and cENR media. Ganglia‐like structures formed in all samples (indicated by arrowheads). Scale bar: 100 μm. (D) Viability and cell death assays were performed using CellTiter‐Glo and Caspase‐1 assays, respectively. Values are presented as mean ± SEM. Statistical analysis was performed using one‐way ANOVA with Sidak's post‐test; ns, not significant (Table S2). Abbreviations: AChE, acetylcholinesterase; cENR, organoid culture medium; CTR, control; ENS, enteric nervous system; RLU, relative light units.

**Table S1:** List of primers for RT‐qPCR.

**Table S2:** Figure panel data and statistical specifications.

**Table S3:** Proteomics data of colonic organoids.

## Data Availability

All data are available in the main text or Supporting Information files.
