# Supplementary material for: Interplay of the ENS and Microbiota With Murine Gut Epithelium‐Derived Organoids in Aging
Source: Aging Cell. 2026 Jul 3;25(7):e70608. doi: 10.1111/acel.70608 (PMC13329257; doi:10.1111/acel.70608)
Supplement: Supplementary file 1 — Figure S1: Representative microinjection of propidium iodide dye into organoid. Images of organoids are shown in bright‐field (upper panel) or fluorescence (middle panel) or merge (lower panel). Images taken before and after (0, 1‐ or 3‐days) microinjection of propidium iodide dye with Nanoject III. Increasing thickness of organoid lumen indicated cell regeneration upon injection. Scale bar: 50 μm. Figure S2: Reproducibility scatterplots for organoids derived from young SAMR1 and SAMP8. Scatterplot matrices depicting high reproducibility between replicates of the same batch (replicates 1 & 2 or replicates 3 & 4) based on log10‐transformed, normalized, and corrected protein intensities. Data are shown for organoids derived from young male (A) and female (B) SAMR1, and male (C) and female (D) SAMP8 mice. A batch effect is observed between the two batches of samples (replicates 1 & 2 vs. replicates 3 & 4). Abbreviations: F, female; M, male. Figure S3: Reproducibility scatterplots for organoids derived from aged SAMR1 and SAMP8.Scatterplot matrices depicting high reproducibility between replicates of the same batch (replicates 1 & 2 or replicates 3 & 4) based on log10‐transformed, normalized, and corrected protein intensities. Data are shown for organoids derived from aged male (A) and female (B) SAMR1, and male (C) and female (D) SAMP8 mice. A batch effect is observed between the two batches of samples (replicates 1 & 2 vs. replicates 3 & 4). Abbreviations: F, female; M, male. Figure S4: Principal component analysis plot of replicates for proteomics analysis. Principal component analysis of the 500 most variable proteins based on intensities following normalization by median‐centering. A batch effect is observed between the two batches of replicates. Abbreviation: PC, principal component. Figure S5: Validation of antibodies against Fut2 and Fut4 for Western blot analysis. To validate the antibodies against Fut2 and Fut4 used for Western blotting, murine tissues with kn [file ACEL-25-e70608-s003.docx]

**Supporting Information**

**Production of Noggin- and R-spondin-conditioned media**

The Noggin- and R-spondin-producing HEK cell lines used were as follows: 1) HEK293 T-mNoggin-Fc (HEK-Noggin), for which permission of usage was obtained from the MTA, Hubrecht Institute, Netherlands, is resistant to Geneticin disulfate (G418) (Carl Roth) and 2) Cultrex HA-R-Spondin1-Fc 293T (HEK-R-spondin) (R&D systems) is resistant to Zeocin (Invitrogen).

*HEK cell culture*

The vials of cryopreserved Noggin- and R-spondin-producing HEK cells were thawed in a 37°C water bath for 2 min. The thawed cells were transferred into separated cell culture dishes with 20 mL of pre-warmed HEK growth medium, containing DMEM high glucose (Thermo Fisher Scientific), 100 U/mL Penicillin-Streptomycin (Thermo Fisher Scientific), 2 mM L-glutamine (Thermo Fisher Scientific), 10% Fetal Bovine Serum (Thermo Fisher Scientific), with corresponding antibiotics (300 µg/mL Zeocin for HEK-R-spondin cells and 500 µg/mL G418 for HEK-Noggin cells). The cryovials were washed with 1 mL of pre-warmed HEK growth medium each, which was added to the dishes. The cells were well-distributed in the dishes and placed in a humidified cell incubator at 37°C and 5% CO_2_ afterward. The HEK-R-spondin cells could be passaged a day after thawing and every 2 days after the previous passage. For the HEK-Noggin, cells could be passaged two days after thawing and every 3–4 days after the previous passage. Cells were passaged upon reaching 80% confluency, according to the protocol described following. The medium was discarded, and the cells were washed with 5 mL of pre-warmed PBS. Subsequently, 3 mL of trypsin (Biological Industries) were added, and cells were incubated for 3 min at 37°C, 95% humidity, and 5% CO_2_ incubator. The trypsin reaction was stopped by adding 5 mL of HEK growth medium. Following resuspension, the cells were transferred to a 50 mL tube and centrifuged at 500 × g for 5 min at RT. The cell supernatant was removed, and the cell pellets were suspended in HEK growth medium with corresponding antibiotics to achieve six new dishes of cells (10mL cell suspension per dish). Cell culture dishes were placed in the incubator.

*Collection of conditioned media*

For the production and collection of Noggin and R-spondin conditioned media, the two HEK-based cell lines were cultured until passage number three to remain stable. At passage number four, cells were split the same as described above but added with HEK growth medium without Zeocin/G418 and cultured until reaching confluency. At this point, the HEK growth medium was aspirated completely and 16.2 mL of pre-warmed AD-DF+++ medium were carefully added to each dish. Cells were maintained in culture for 7 days. Then the cell supernatant was collected and centrifuged at 500 × g for 5 min followed by filtering through a 0.2 µm filter to remove any remaining cells or cell fragments. The collected conditioned media were aliquoted and stored at -20°C for preparing the organoid culture medium.

**Proteomics procedure and data processing**

*Enzymatic protein digestion and TMT labelling*

All samples were processed using the SP3 approach (Hughes et al., 2019). The proteins were reduced in DTT and alkylated by iodoacetamide in the dark. Enzymatic protein digestion was performed using trypsin overnight at 37°C. The resultant peptide solution was purified by solid phase extraction in C18 StageTips (Rappsilber, Ishihama, & Mann, 2003).

The desalted peptides were eluted in 80% acetonitrile. The volume of the peptide solution was then reduced in a centrifugal evaporator before adding 100 mM triethylammonium bicarbonate (TEAB). Afterwards, peptides were labelled using 16-plex TMTPro reagents, according to manufacturer’s instructions (Thermo Fisher Scientific). To accommodate a total number of 32 samples (8 conditions × 4 replicates), the 4 replicates of each condition were split into 2 batches and combined into two 16-plex peptide mixtures.

*Tip-based high-pH reversed phase peptide fractionation*

The TMT-labelled peptide mixture was first desalted on C18 StageTip (Empore, 3M) and then fractionated on the tip in 10 mM TEAB at pH 8 sequentially at the following concentrations of acetonitrile: 3%, 5%, 7%, 9%, 12%, 15%, 18%, 21%, 25%, 30%, 60%. With the exception of the faction at 15% acetonitrile, the early and late fractions were further concatenated as the following combinations: 3% + 18%, 5% + 21%, 7% + 25%, 9% + 30%, 12% + 60%. In total, 6 peptide fractions per sample were prepared and dried to completeness in a centrifugal evaporator at 45 °C. The dried peptides were reconstituted in 80% acetonitrile, 0.1% formic acid. After the acetonitrile was evaporated in a centrifugal evaporator, the peptides were acidified by adding 0.1% formic acid.

*Liquid chromatography tandem mass spectrometry*

Peptides were separated via an in-house packed 45-cm analytical column (inner diameter: 75 μm; ReproSil-Pur 120 C18-AQ 1.9-μm silica particles, Dr. Maisch GmbH) on a Vanquish Neo UHPLC system (Thermo Fisher Scientific). The online reversed-phase chromatography separation was conducted through a 70-min non-linear gradient of 1.6-34.4% acetonitrile in 0.1% formic acid at a nanoflow rate of 300 nL/min. The eluted peptides were sprayed directly by electrospray ionization into an Orbitrap Astral mass spectrometer (Thermo Fisher Scientific). Mass spectrometry was conducted in data-dependent acquisition mode using a top50 method with one full scan in the Orbitrap analyzer (scan range: 350 to 1,500 m/z; resolution: 120,000, target value: 3 × 106, maximum injection time: 20 ms, precursor fit purity: 50% threshold and 1.2 m/z window) followed by 50 fragment scans in the Astral analyzer via higher energy collision dissociation (HCD; normalised collision energy: 33%, scan range: 110 to 1,100 m/z, target value: 1 × 104, maximum injection time: 10 ms, isolation window: 0.5 m/z, TMT option: on). Precursor ions of unassigned, +1 or higher than +6 charge state were rejected. Additionally, precursor ions already isolated for fragmentation were dynamically excluded for 15 s.

*Mass spectrometry data processing*

Mass spectrometry raw data files were processed using MaxQuant software (version 2.4.2.0) (Cox & Mann, 2008). MS/MS mass spectra were searched using Andromeda search engine (Cox et al., 2011) against a target-decoy database containing the forward and reverse protein sequences of UniProt M. musculus reference proteome (release 2023_01; 63,606 entries) and a default list of common contaminants. Corresponding 16-plex labels were assigned including the correction factors for isotope impurity provided by the manufacturer. Carbamidomethylation of cysteine was set as fixed modification. Methionine oxidation and protein N-terminal acetylation were chosen as variable modifications. Trypsin/P specificity was assigned. A maximum of 2 missed cleavages were tolerated. The minimum peptide length was set to be 7 amino acids. The “second peptides” search was switched on. The “match between runs” function was also activated (Yu, Kyriakidou, & Cox, 2020). False discovery rate was set to 1% at both peptide and protein levels. Both the unique and razor peptides were used for protein quantification.

**Western blotting**

To validate the antibodies against Fut2 and Fut4 used for Western blotting, murine tissues with known expression status, as well as colonic organoids treated with fucose (substrate of either enzyme), were analyzed. For this purpose, colon, heart, and bone marrow tissues were isolated from a 3-month-old wild-type C57BL/6J mouse.

*Bone marrow isolation*

Bone marrow was collected from the hind limbs. Briefly, skin was removed from both hind limbs, and surrounding muscle tissue was carefully dissected away using forceps to expose the bones. Both epiphyses were trimmed to open the marrow cavity, and the bones were immediately placed in ice-cold PBS. A small hole was made at the bottom of a 0.5 mL microcentrifuge tube, and each bone was positioned with the opened marrow cavity facing downward inside the tube. The 0.5 mL tube was then placed into a 1.5 mL collection tube and centrifuged at 5,500 × g for 30 s at 4°C. The supernatant was discarded, and the resulting pellet containing bone marrow cells was collected.

*Organoid treatment with fucose*

Colonic organoids derived from 3-month-old female SAMR1 mice at day 4 after passaging were treated with 5 mg/mL fucose (Sigma) in cENR medium for 3 days. Organoids at day 7 were subsequently harvested as described in the Materials and Methods section.

*Protein extraction*

Colon, heart, bone marrow samples, and fucose-treated organoids were homogenized in RIPA buffer (25 mM Tris-HCl, pH 8, 1 mM EDTA, 150 mM NaCl, 1% Nonidet P-40, 0.5% sodium deoxycholate, 0.1% SDS, and 1× proteinase inhibitor cocktail) using a metal homogenizer (Fluke device, Appliance ID 0966) for 2 min at speed 1 on ice. Lysates were then centrifuged at 16,000 × g for 5 min at 4°C, and the supernatants were collected for downstream Western blot analysis.

*Western blotting*

15 µg of lysates of colon, heart, and bone marrow while 15 µg and 25 µg of protein extracted from organoids as described above were loaded onto a 10% SDS-PAA gel and transferred to a nitrocellulose membrane by tank blot method. The primary antibodies were as follows: anti-Fut2 (Biorbyt, orb 156968, dilution 1:1000 in 5% nonfat dried milk blocking solution (ITW Reagents)), and anti-Fut4 (Biorbyt, orb627154, dilution 1:1000 in 5% nonfat dried milk blocking solution (ITW Reagents)). Quantification was performed by using appropriate HRP-conjugated secondary antibodies and SuperSignal™ West Femto Maximum Sensitivity Substrate (Thermo Fisher Scientific), with signal acquisition carried out using a Lumi Imager F1 (Boehringer Mannheim).

**Cell viability and Caspase assay**

To evaluate effect of the ENS and the organoid culture media on the ENS growth capacity and activity, the LMMP was isolated from young and aged female SAMR1 mice (n = 3 animals per age group, with n = 4 tissue pieces per animal, for each medium condition). The tissue was embedded in BME in a white 96‐well plate (Greiner Bio‐One) and cultured either in standard ENS growth medium (abbreviated as ENS medium; Neurobasal-A (Thermo Fisher Scientific) supplemented with 10 ng/mL GDNF (Origene), 1 × B-27, 1% FBS, 2 mM L-Glutamine, and 100 U/mL Penicillin-Streptomycin) or in cENR medium, which was used for organoid culture as well as for organoid-ENS co-culture. After 7 days in culture, LMMP samples from each condition were removed from the wells to perform the AChE assay as described in the Materials and Methods section. The remaining cells that had migrated from the LMMP tissue were maintained within the BME dome in their respective media, with experiments typically initiated on day 14 of culture.

For cell viability assessment, 50 µL of CellTiter-Glo 3D reagent (Promega Corporation) was added to each well containing 50 µL of culture medium, followed by agitation for 5 min at 300 rpm on a plate shaker (LLG Labware) at RT. The plate was then incubated at RT, protected from light, for 25 min.

For cell death analysis, 50 µL of Caspase-1 reagent (Promega Corporation) was added to each well containing 50 µL of culture medium and agitated for 30 s at 300 rpm on a plate shaker (LLG Labware) at RT. The plate was subsequently incubated at RT, protected from light, for 90 min.

Luminescence was measured using a FLUOstar Optima microplate reader (BMG Labtech) with a gain setting of 3650, recording 10 measurements at 0.5 s intervals. Signals were expressed as relative light units (RLU), normalized to the control condition (ENS medium), and presented as a percentage of control (CTR).

**References**

Cox, J., & Mann, M. (2008). MaxQuant enables high peptide identification rates, individualized p.p.b.-range mass accuracies and proteome-wide protein quantification. *Nature Biotechnology, 26*(12), 1367-1372. doi:10.1038/nbt.1511

Cox, J., Neuhauser, N., Michalski, A., Scheltema, R. A., Olsen, J. V., & Mann, M. (2011). Andromeda: A Peptide Search Engine Integrated into the MaxQuant Environment. *Journal of Proteome Research, 10*(4), 1794-1805. doi:10.1021/pr101065j

Hughes, C. S., Moggridge, S., Müller, T., Sorensen, P. H., Morin, G. B., & Krijgsveld, J. (2019). Single-pot, solid-phase-enhanced sample preparation for proteomics experiments. *Nature Protocols, 14*(1), 68-+. doi:10.1038/s41596-018-0082-x

Rappsilber, J., Ishihama, Y., & Mann, M. (2003). Stop and go extraction tips for matrix-assisted laser desorption/ionization, nanoelectrospray, and LC/MS sample pretreatment in proteomics. *Analytical Chemistry, 75*(3), 663-670. doi:10.1021/ac026117i

Yu, S. H., Kyriakidou, P., & Cox, J. (2020). Isobaric Matching between Runs and Novel PSM-Level Normalization in MaxQuant Strongly Improve Reporter Ion-Based Quantification. *Journal of Proteome Research, 19*(10), 3945-3954. doi:10.1021/acs.jproteome.0c00209

**Supplementary Figures**

**
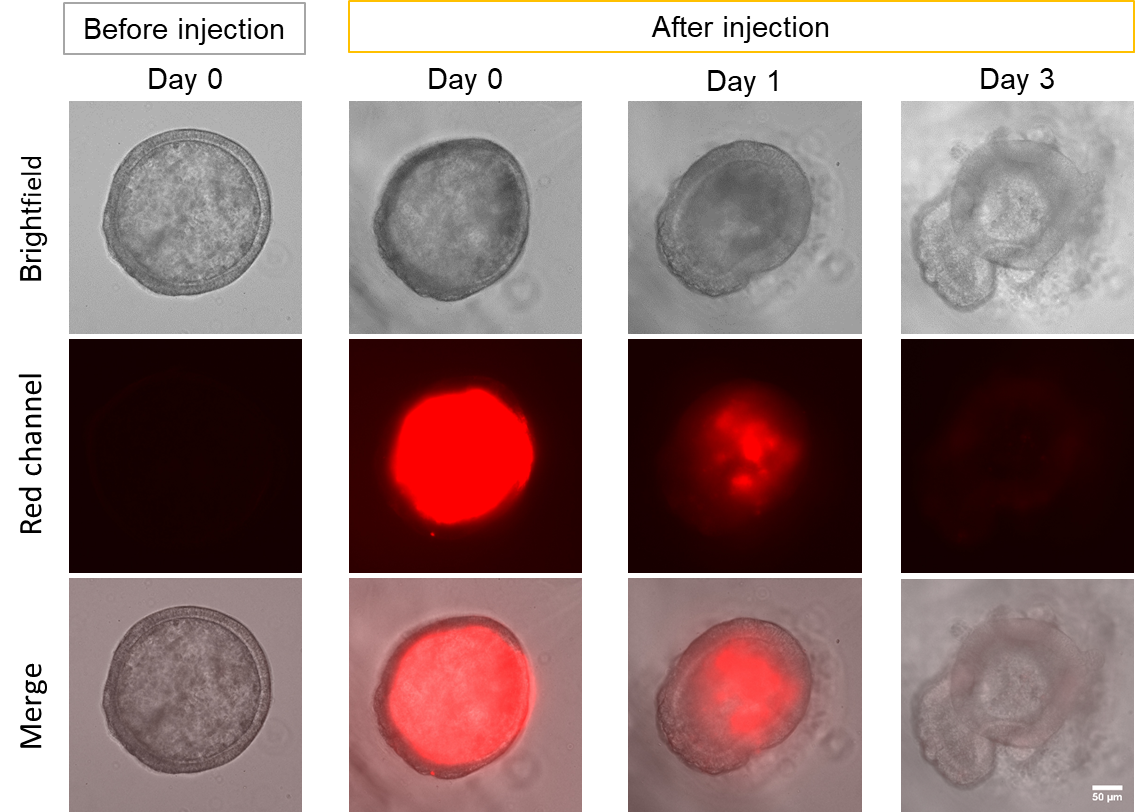
**

***Figure S1. Representative microinjection of propidium iodide dye into organoid.***

*Images of organoids are shown in bright-field (upper panel) or fluorescence (middle panel) or merge (lower panel). Images taken before and after (0, 1- or 3-days) microinjection of propidium iodide dye with Nanoject III. Increasing thickness of organoid lumen indicated cell regeneration upon injection. Scale bar: 50 µm.*


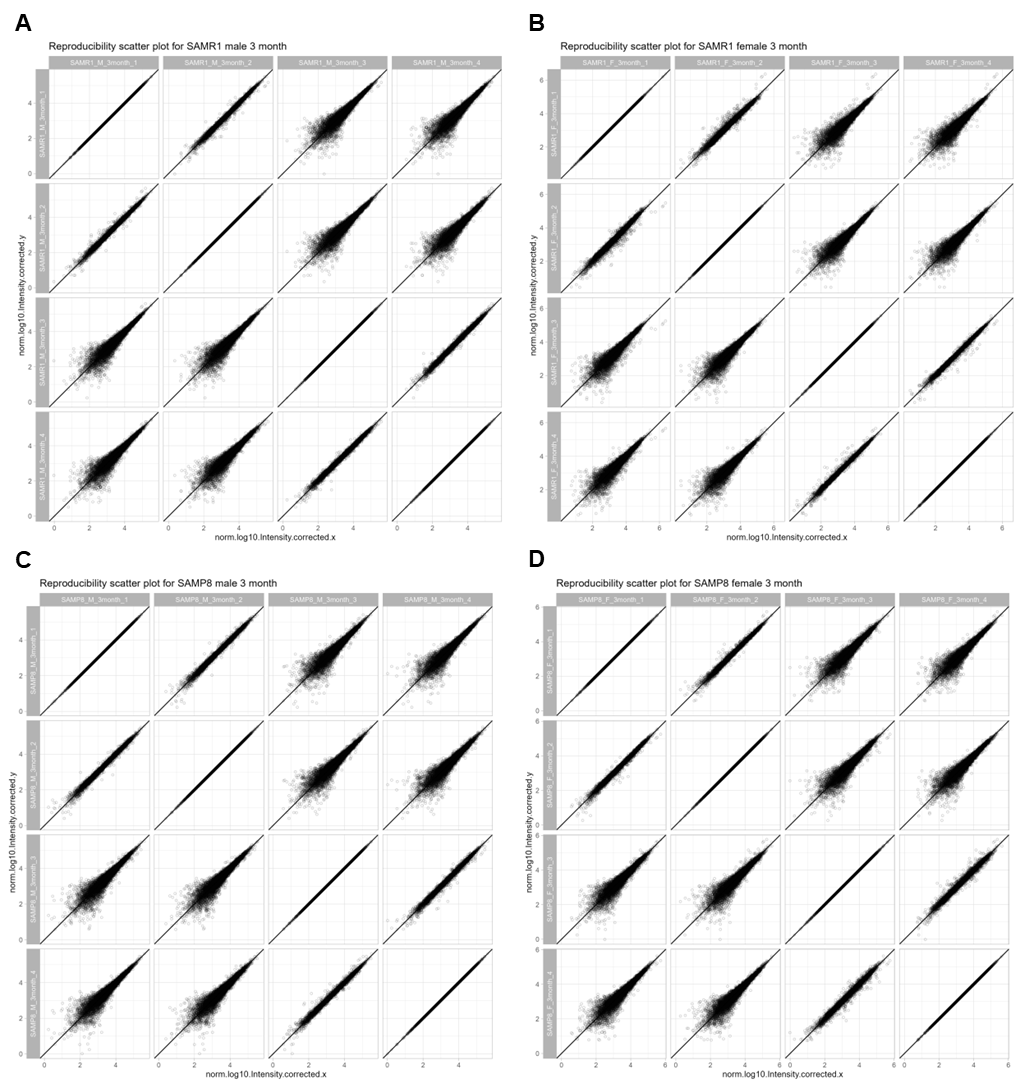


***Figure S2. Reproducibility scatterplots for organoids derived from young SAMR1 and SAMP8.***

*Scatterplot matrices depicting high reproducibility between replicates of the same batch (replicates 1 & 2 or replicates 3 & 4) based on log10-transformed, normalized, and corrected protein intensities. Data are shown for organoids derived from young male (A) and female (B) SAMR1, and male (C) and female (D) SAMP8 mice. A batch effect is observed between the two batches of samples (replicates 1 & 2 versus replicates 3 & 4).* *Abbreviations: M, male; F, female.*


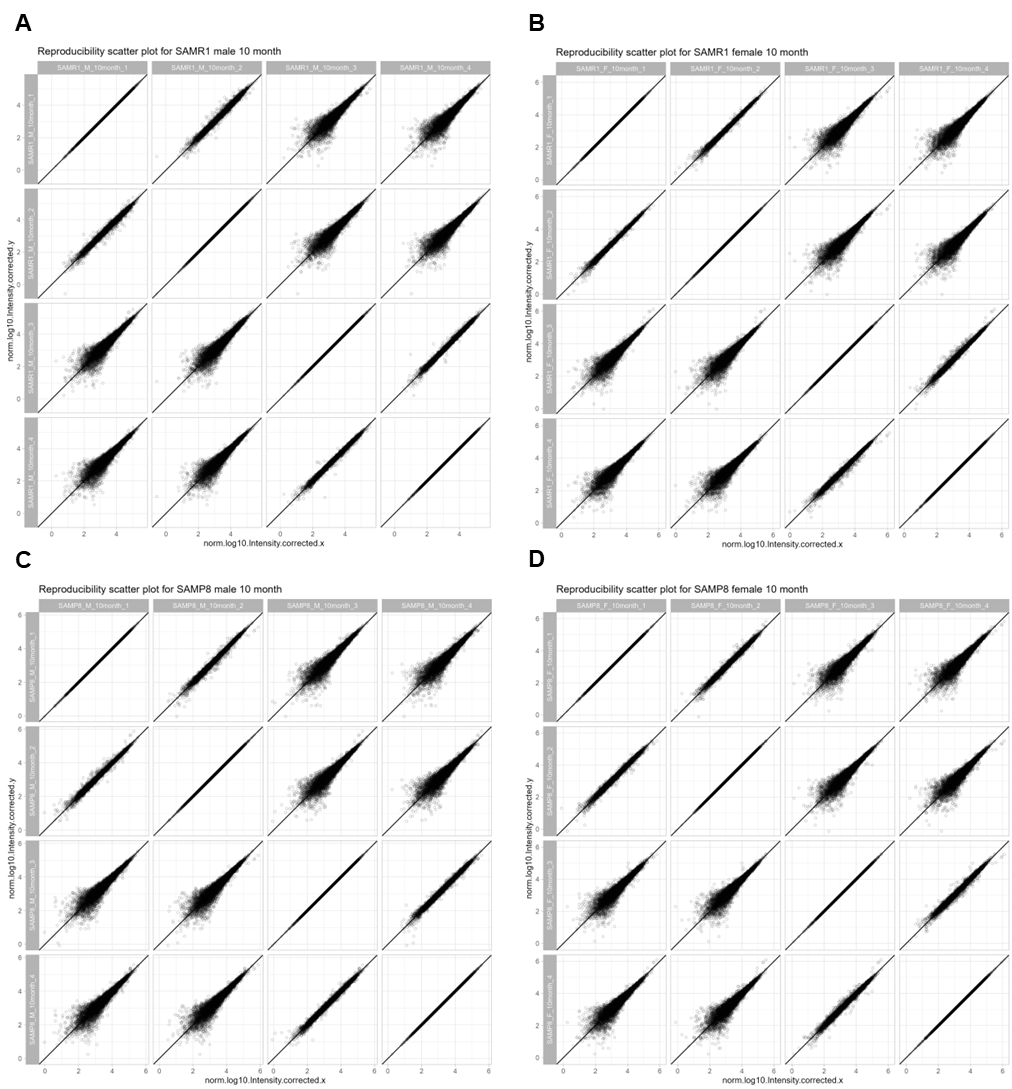


***Figure S3. Reproducibility scatterplots for organoids derived from aged SAMR1 and SAMP8.***

*Scatterplot matrices depicting high reproducibility between replicates of the same batch (replicates 1 & 2 or replicates 3 & 4) based on log10-transformed, normalized, and corrected protein intensities. Data are shown for organoids derived from aged male (A) and female (B) SAMR1, and male (C) and female (D) SAMP8 mice. A batch effect is observed between the two batches of samples (replicates 1 & 2 versus replicates 3 & 4).* *Abbreviations: M, male; F, female.*


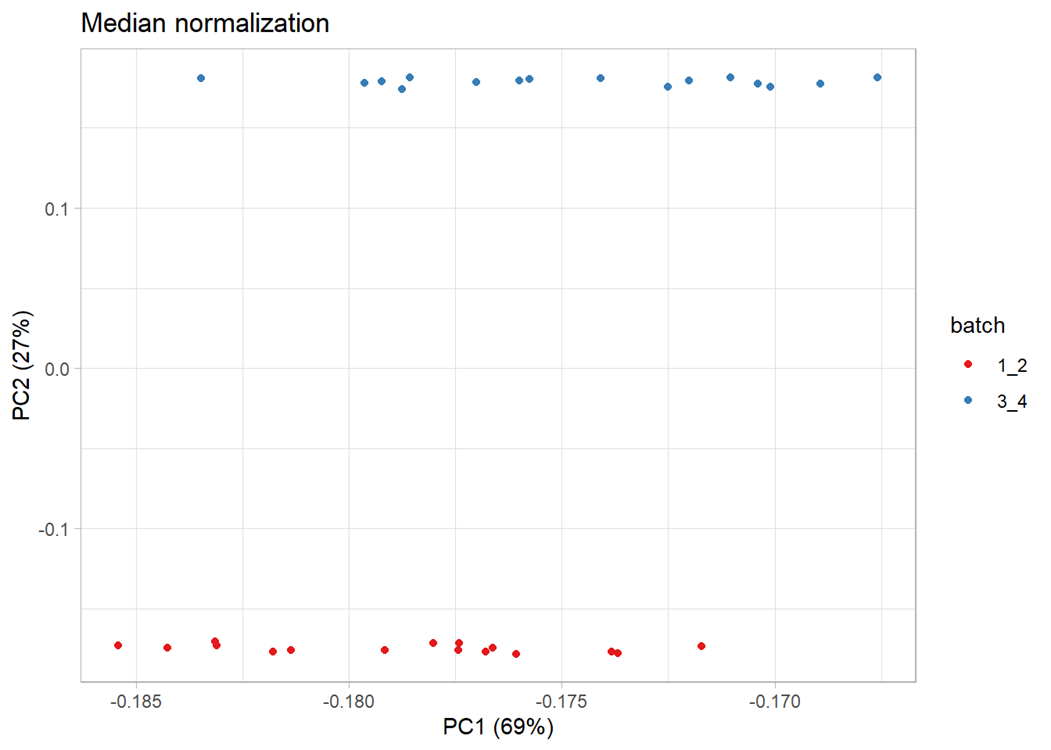


***Figure S4. Principal component analysis plot of replicates for proteomics analysis.***

*Principal component analysis of the 500 most variable proteins based on intensities following normalization by median-centering. A batch effect is observed between the two batches of replicates. Abbreviations: PC, principal component.*


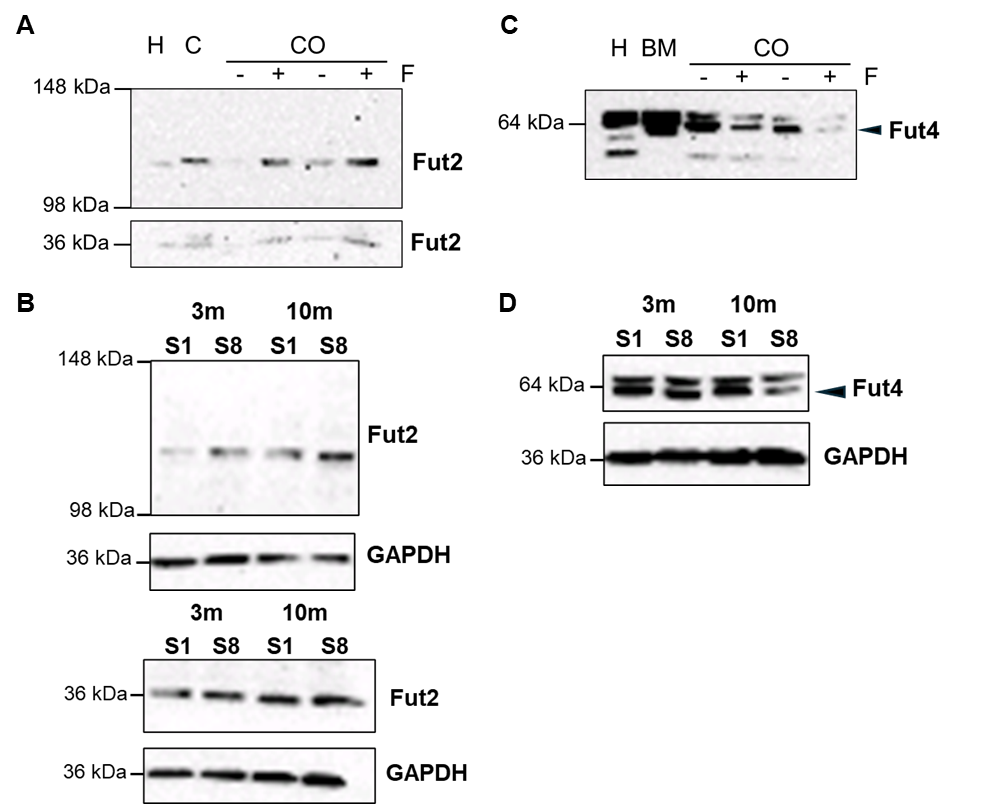


***Figure S5. Validation of antibodies against Fut2 and Fut4 for Western blot analysis.***

*To validate the antibodies against Fut2 and Fut4 used for Western blotting, murine tissues with known expression status, as well as colonic organoids treated with fucose (substrate of either enzyme), were analyzed. (A) Western blot analysis of Fut2 in murine heart (negative control), colon (positive control), and colonic organoids derived from young female SAMR1 mice without (–) and with (+) fucose treatment. (B) Representative Western blot of Fut2 in colonic organoids derived from young and aged male SAMR1 and SAMP8 mice, related to Fig. 5D. (C) Western blot analysis of Fut4 in murine heart (negative control), bone marrow (positive control), and colonic organoids derived from young female SAMR1 mice without (–) and with (+) fucose treatment. (D) Representative Western blot of Fut4 in colonic organoids derived from young and aged female SAMR1 and SAMP8 mice, related to Fig. 5E. GAPDH served as a loading control. Abbreviations: BM: bone marrow; C, colon; CO, colonic organoid; F, fucose; H, heart; m, month; S1, SAMR1; S8, SAMP8; –, without; +, with.*

*
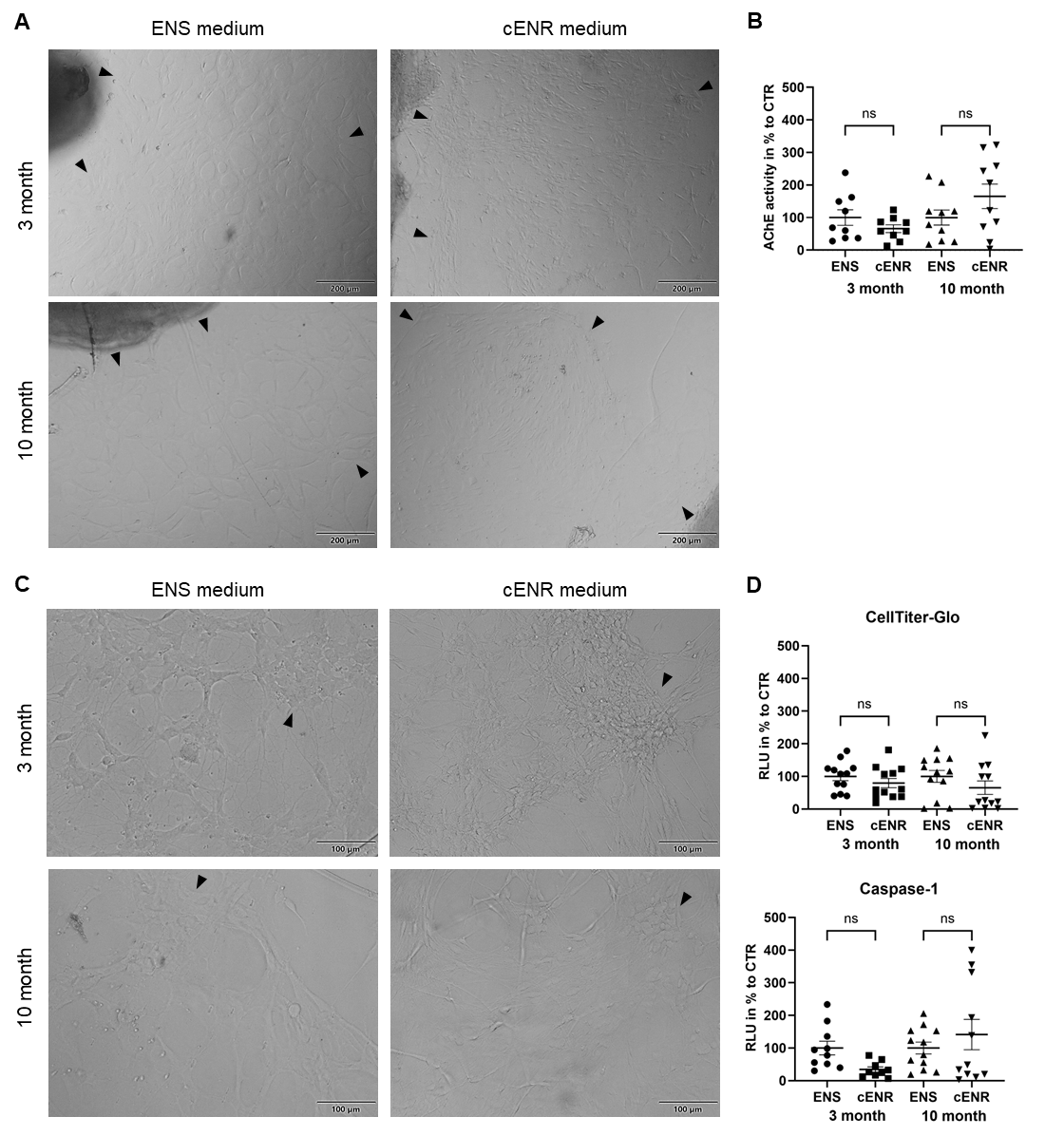
*

***Figure S6. Comparison of ENS and organoid culture medium on ENS growth capacity and activity.***

*To compare the impact of the ENS and the organoid culture medium on ENS growth capacity and activity, LMMPs were cultured in standard ENS growth medium (ENS medium) and in cENR medium, which was used for organoid culture as well as for organoid-ENS co-culture. For this purpose, samples were isolated from young and aged female SAMR1 mice (n = 3 animals per age group, with n = 4 technical replicates per animal). (A) Images taken after 7 days of LMMP culture in ENS and cENR medium. Cells grew and migrated from LMMP tissue derived from animals at both ages in both media (indicated by arrowheads). Scale bar: 200 µm. (B) AChE activity of the ENS in both culture media. (C) Images taken after 14 days of LMMP culture in ENS and cENR media. Ganglia-like structures formed in all samples (indicated by arrowheads). Scale bar: 100 µm. (D) Viability and cell death assays were performed using CellTiter-Glo and Caspase-1 assays, respectively. Values are presented as mean ± SEM. Statistical analysis was performed using one-way ANOVA with Sidak’s post-test; ns, not significant (Table S2). Abbreviations: AChE, acetylcholinesterase; CTR, control; ENS, enteric nervous system; cENR, organoid culture medium; RLU, relative light units.*

**Supplementary Tables**

***Table S1. List of primers for RT-qPCR***

***Table S2. Figure panel data and statistical specifications***

***Table S3. Proteomics data of colonic organoids***
